# Cultural differences in vocal emotion recognition: a behavioural and skin conductance study in Portugal and Guinea-Bissau

**DOI:** 10.1007/s00426-021-01498-2

**Published:** 2021-03-15

**Authors:** Gonçalo Cosme, Vânia Tavares, Guilherme Nobre, César Lima, Rui Sá, Pedro Rosa, Diana Prata

**Affiliations:** 1 Instituto de Biofísica e Engenharia Biomédica, Faculdade de Ciências da Universidade de Lisboa, Campo Grande 016, 1749-016 Lisboa, Portugal; 2 Faculdade de Medicina, Universidade de Lisboa, Lisboa, Portugal; 3 Centro de Investigação e Intervenção Social, Instituto Universitário de Lisboa (ISCTE-IUL), CIS-IUL, Lisboa, Portugal; 4 CAPP-Centre for Public Administration & Public Policies, ISCSP, Universidade de Lisboa, Lisboa, Portugal; 5 Environmental Sciences Department, Universidade Lusófona da Guiné, Bissau, Guinea-Bissau; 6 HEI-LAB: Human-Environment Interaction Lab/Universidade Lusófona de Humanidades e Tecnologias, Lisboa, Portugal; 7 Department of Neuroimaging, Institute of Psychiatry, Psychology and Neuroscience, King’s College London, London, UK

## Abstract

Cross-cultural studies of emotion recognition in nonverbal vocalizations not only support the universality hypothesis for its innate features, but also an in-group advantage for culture-dependent features. Nevertheless, in such studies, differences in socio-economic-educational status have not always been accounted for, with idiomatic translation of emotional concepts being a limitation, and the underlying psychophysiological mechanisms still un-researched. We set out to investigate whether native residents from Guinea-Bissau (West African culture) and Portugal (Western European culture)—matched for socio-economic-educational status, sex and language—varied in behavioural and autonomic system response during emotion recognition of nonverbal vocalizations from Portuguese individuals. Overall, Guinea–Bissauans (as out-group) responded significantly less accurately (corrected *p* < .05), slower, and showed a trend for higher concomitant skin conductance, compared to Portuguese (as in-group)—findings which may indicate a higher cognitive effort stemming from higher difficulty in discerning emotions from another culture. Specifically, accuracy differences were particularly found for pleasure, amusement, and anger, rather than for sadness, relief or fear. Nevertheless, both cultures recognized all emotions above-chance level. The perceived authenticity, measured for the first time in nonverbal cross-cultural research, in the same vocalizations, retrieved no difference between cultures in accuracy, but still a slower response from the out-group. Lastly, we provide—to our knowledge—a first account of how skin conductance response varies between nonverbally vocalized emotions, with significant differences (*p* < .05). In sum, we provide behavioural and psychophysiological data, demographically and language-matched, that supports cultural and emotion effects on vocal emotion recognition and perceived authenticity, as well as the universality hypothesis.

**Supplementary Information:**

The online version contains supplementary material available at 10.1007/s00426-021-01498-2.

## Introduction

Emotion recognition is a crucial cognitive process in human social interactions as it allows for the adequate response to relevant social stimuli (Briefer 2012). Since basic emotions have a fixed set of neurobiological markers (Tracy and Randles 2011), according to the universality hypothesis, they are recognized above-chance level across cultures. This recognition ability in humans is strongly driven by innate factors (Elfenbein and Ambady 2002b; Gendron et al. 2014; Tracy and Randles 2011). However, a meta-analysis of 97 studies on emotion recognition within and across cultures (Elfenbein and Ambady 2002b), not only supported the universality model but also a nature–nurture interactionist one whereby the cultural in-group is at an advantage. That is, while emotion display share basic attributes across cultures (in this case, nationalities) suggesting a biological underpin in display and recognition, other attributes are more accurately recognized when judged by members of the same culture of the ones expressing the emotion (i.e. in-group). Indeed, the universality model was supported by evidence of above-chance level emotion recognition for all cultures (for most emotions, particularly if negative) (Cordaro et al. 2016; Jürgens et al. 2013; Koeda et al. 2013; Laukka et al. 2013; Sauter et al. 2010), and cultural effects, including those of ‘in-group advantage’, by evidence of performance differences between cultures (in-group being better than out-group) (Elfenbein and Ambady 2002b; Jürgens et al. 2013; Mesquita and Frijda 1992; Sauter 2010). Additional evidence has demonstrated modulation specifically by language and cultural knowledge (Barrett et al. 2011; Gendron et al. 2014; Jack et al. 2009; Sauter et al. 2010; Wilson-Mendenhall et al. 2011). However, most studies have neglected differences in socio-economic and educational status (Niedenthal et al. 2017) between cultures (with exceptions (Cordaro et al. 2016)), which could be over-estimating the effect of culture, namely the general better performance of Westerners in emotion recognition.

Although most emotion recognition studies use facial expressions as stimuli, nonverbal vocalizations are the most frequently used cues to infer emotional states (Planalp 1996) and may be as efficient as facial expressions or speech prosody, when context is absent. Like facial expressions, nonverbal vocal cues are thought to be a primitive (Laukka et al. 2013) and universal form of communication (Sauter et al. 2010; Scherer et al. 2001), and the in-group advantage in their recognition has also been found (Elfenbein and Ambady 2002b) just as in facial emotion recognition studies (Elfenbein and Ambady 2002b; Haidt and Keltner 1999).

There have been two reports on the impact of culture on emotion recognition in nonverbal vocalizations with use of a context-driven paradigm. In such studies, the participants read or listen to a sentence before the stimuli presentation and then decide which emotional label matches the contextual sentence and stimuli. In the first, English nationals outperformed Himba semi-nomads for both English and Himba nonverbal vocalizations of basic emotions. Education differences are a possible underlying explanation for these results as discussed by the authors. Yet, each culture performed better for displays of their own culture—there was a significant interaction between the culture of the decoder and the culture of the stimuli producer, evidence of in-group advantage (Sauter et al. 2010). By observing, cross-culturally, above-chance level recognition in anger, disgust, fear, sadness, surprise and amusement, this work supports their universal recognition. However, it did not support the universal recognition of the positive emotions of achievement and pleasure as Himba participants did not perform above-chance level for these English nonverbal vocalizations. Performing emotion recognition studies with a preliterate culture has several drawbacks such as the bewilderment and anxiety due to unfamiliarity to the research equipment (i.e. a computer), language, task instructions, cognitive task demands or the research setting. These factors impair the assessment of sensitive behavioural measures like, for example, response latency. The other study also contributed to understanding why emotions are expressed and recognized differently across cultures by studying the recognition rates of 16 emotions in 10 globalized cultures (Cordaro et al. 2016). The authors suggest these differences may exist because each particular emotion is valued differently between cultures, and its nonverbal expression may even have different ‘accents’ (i.e. cultural specificity). Using nonverbal vocalizations of native English speakers, decoders from South Korea and India could not perform above-chance level for desire (for food) and surprise, respectively. Despite these results, the same study reports strong evidence for the universal recognition of sadness, disgust, fear, awe, amusement, pain and contentment by observing multiple cultures recognizing these emotions above-chance level. Yet, it is unclear from what English-speaking culture the nonverbal vocalizations originated from, which makes it hard to make inferences on cultural effects. Nevertheless, although providing a more ‘ecological’ paradigm, context-driven tasks as these, entail unnecessary noise (given that several more words need to undergo translation and potential connotation alteration), potentially leading to an overestimation of cultural differences.

Since the 2002 meta-analysis (Elfenbein and Ambady 2002b), two studies have used nonverbal vocalizations with a context-free paradigm. One study used English natives’ vocalizations and reported Himba participants only recognizing amusement significantly above-chance level, whereas U.S. participants did for all emotions (amusement, anger, disgust, fear, relief, sadness, sensory pleasure, surprise and triumph) (Gendron et al. 2014). In addition, it reports a main effect of cultural group where US participants showed statistically significant better performance when compared to Himbas (not specified per emotion). This study also reported similar results when employing the same task but this time with a contextual story. The authors argue that performance in emotion recognition tasks is dependent on the context given to participants (Cordaro et al. 2016), and that discrete emotion recognition is confounded by valence perception. These results are conflicting with the universality hypothesis as it was expected that all emotions would be recognized above-chance level in both cultures. However, these findings might be confounded by socio-economical and educational status differences between both cultures, and particularly of language, given its participants free-labelled the emotions (i.e. the participants do not have emotion labels to choose from). The second study reported that Swedish participants recognized anger, contempt, disgust, fear, happiness, sadness, surprise, interest, lust, relief, serenity and positive surprise from USA, India, Kenya and Singapore speakers at above-chance level, but not for distress, guilt, shame, negative surprise, affection, amusement and pride (Laukka et al. 2013). Interestingly, all six basic emotions (except the more specific negative surprise) were recognized above-chance level for speakers of all cultures, which supports the universality being relatively higher for basic emotions, and these being more ‘hard-wired’ in the human brain.

The universality hypothesis expects basic emotions to be recognized above-chance level across in- or out-groups independent their valence (Elfenbein and Ambady 2002b). Yet, there is previous evidence that negative emotions are particularly less susceptible to cultural effects, such as in-group advantage, than positive emotions (Elfenbein and Ambady 2002b). This is possibly due to the fact that negative emotions are displayed relatively more similarly across mammals and used as signals for a larger social audience, carrying a strong survival-relevant role with the goal of spreading information of danger, such as in the case of anger and fear—although this hypothesis is unclear for sadness. On the other hand, positive emotions would not be as biologically hard-wired for survival, and would rather have a major in-group bonding role (Elfenbein et al. 2007; Fredrickson 2001; Laukka et al. 2013). Indeed, a study using nonverbal vocalizations from both studied cultures (English and Himbas) (Sauter et al. 2010) supports a better fit of the universality hypothesis for negative, rather than positive emotions, by showing Himbas recognized more negative (anger, disgust, fear and sadness) than positive (amusement) emotions vocalized by English speakers above-chance level, whereas English participants recognized all emotions from Himba speakers above-chance level. However, other studies report cultural differences in the recognition of negative emotions, such as anger, disgust and fear (Gendron et al. 2014; Koeda et al. 2013).

Nonverbal vocalizations can be either authentic (spontaneous) or acted (fake) and discriminating between them is an important cognitive empathy skill. Different social outcomes may arise when an emotion is expressed authentically compared to acted. For example, authentic laughter can foster social bonding, and acted laughter may signal deception (Gervais and Wilson 2005; Scott et al. 2014; ten Brinke and Porter 2012). We have previously investigated this emotional vocalization quality having shown that: (1) laughs, for example, perceived as more authentic were also rated as more arousing (Lavan et al. 2016); (2) emotions such as achievement, anger, fear and pleasure were more likely to be perceived as authentic than amusement, disgust and sadness (Anikin and Lima 2017), even if all authentic; (3) when exposed to both authentic and acted stimuli, participants were more accurate in recognizing the authenticity of fear and least accurate in disgust (Anikin and Lima 2017); (4) perceived authenticity (positively) affected emotion recognition accuracy even if all stimuli were acted (Lima et al. 2013), and (5) that valence seems to influence authenticity perception, with positive emotions rated as more authentic than negative ones (Lima et al. 2013), in what pertains to emotion recognition. Cultural specificity in authenticity discrimination has already been reported (between authentic and acted prosodic sounds) (Jürgens et al. 2013), whereby a German in-group outperformed Romanian and Indonesian decoders in anger, fear, joy and sadness. Nevertheless, cultural differences in the perception of authenticity of emotional vocalizations have not yet been researched.

In the present study, we asked whether people are as good (and respond with similar emotional arousal and/or cognitive load—see below) identifying nonverbal acted vocalizations of emotion from their own culture (Portuguese; i.e. ‘in group’) as people from another culture (Guinea-Bissau; i.e. ‘out-group’). Like two previous ones, this study uses a context-free paradigm, but, to our knowledge, it is the first testing the universality hypothesis, and a main effect of culture, using socio-economically, language and educationally matched samples. We tested natives from Portugal and natives from Guinea-Bissau (a Portuguese colony until 1974 (Miller 1975)) in regards to their recognition, and perceived authenticity, of nonverbal acted emotional vocalizations from Portuguese individuals (Lima et al. 2013). These two populations have not yet been researched in cross-cultural emotion studies*.* Participants from both nationalities de facto speak the same, and have the same official language (Portuguese), thus bypassing the need for idiom translation; and are undergoing (or recently graduated in) a university degree in the biomedical field, thus matching as much as possible for socio-economical and educational status in relation to national standards.

In addition, for the first time in cross-cultural emotion recognition research, and altogether in nonverbal emotion recognition research, we complement behavioural measurements with concomitant skin conductance response measurements (SCR; which reflect eccrine sweat gland activity, triggered by acetylcholine release in the sympathetic nervous system (Khalfa et al. 2002)). In particular, we consider a heightened SCR a positive proxy of autonomic sympathetic arousal putatively deriving from cognitive load (Engström et al. 2005; Mehler et al. 2012; Nourbakhsh et al. 2017). This is supported by its positive correlation with pupil dilation (Wang et al. 2018), in turn also a positive proxy for emotional and cognitive load arousal (Peysakhovich et al. 2015; Siegle et al. 2003; Zénon et al. 2014). Herein, we have collected the following SCR measures: latency (i.e. the period between stimuli onset and the first significant deviation), found to be negatively correlated with arousing luminance (Wolfensberger and O’Connor 1967); amplitude (i.e. the degree of deviation or the magnitude of the response), a positive proxy of sympathetic activity in general (Benedek and Kaernbach 2010), facial emotional recognition in particular (Lang et al. 1993; Skwerer et al. 2009) and arousal stemming from cognitive load (MacPherson et al. 2017; Nourbakhsh et al. 2017; Shi et al. 2007); rise time (i.e. duration until amplitude peak) which has been negatively correlated with reactivity to auditory stimuli (Boucsein 2012; Venables et al. 1980); and SCR percentage (i.e. the percentage of stimuli that actually elicited SCR amplitude) as it is positively correlated with facial emotional arousal (Skwerer et al. 2009). Although not yet researched in nonverbal emotion vocalizations, different emotions have been shown to trigger different SCR patterns in prosodic vocalizations; for example, anger, happiness and sadness led to higher SCR amplitude than neutral sounds (Petrone et al. 2016), and anger, can trigger a shorter SCR latency, probably due to the importance of signalling a potential danger (Petrone et al. 2016).

We expected to find a universality effect such that all emotions would be recognized above-chance level by both cultures—based on previously reported evidence (Koeda et al. 2013)—as well as a main effect of nationality such that participants from Guinea-Bissau: (1) will be less accurate, and slower, than the Portuguese in emotion recognition of nonverbal vocalizations from Portuguese, particularly in positive (vs. negative) emotions (Koeda et al. 2013; Laukka et al. 2013; Sauter et al. 2010); (2) show concomitant SCR proxies for higher autonomic system arousal and/or cognitive load such as lower latency, higher amplitude and percentage of SCR and longer rise times (Dawson, Schell, & Filion, 2007); and (3) will be more susceptible than Portuguese to perceive these acted stimuli as authentic (Jürgens et al. 2013). We explore how these behavioural and physiological effects vary between emotions, without a priori hypotheses for such interaction effects; and we examine for the first time how different nonverbally vocalized emotions differ in SCR. Lastly, in support of these hypotheses and replication to our previous finding, we expect vocalizations that were perceived as more authentic to entice higher emotion recognition accuracy (Lima et al. 2013), and that both are negatively associated with the corresponding response latencies.

## Materials and methods

### Participants

Thirty-eight West Africans native from Guinea-Bissau (26 males, 12 females) and 35 Western European native from Portugal (24 males, 11 females) were recruited at a university campus (from nursing, medicine, and biology courses) by word-of-mouth and posters. They voluntarily participated in this study under informed consent (Ethics approval from Universidade Lusófona da Guiné, Guinea-Bissau—reference ULG 01/2016CA, and Centro Hospitalar Lisboa Norte, Portugal—reference 192/16). Inclusion criteria were being a university student between 18 and 45 years old in a biomedical field, for socio-educational-economic in-culture homogeneity and cross-cultural matching, besides sex; with no chronic or acute illness, or past head injury followed by loss of consciousness; and consumption of less than 28 alcohol units per week or 5 cigarettes a day in the last 6 months. All participants were also confirmed to have a hearing ability sufficient for the quiet conversation taking place during the session. Four participants were excluded after reporting head injury with loss of consciousness in the last year, plus one due to not understanding task instructions, and three due to data being mis-recorded, resulting in 33 Guinea–Bissauan and 32 Portuguese. None of the females was pregnant and all participants performed the tasks with their dominant hand. Alcohol and drug use were statistical and significantly higher in Guinea–Bissauan than Portuguese males. For detailed demographic characteristics and sample comparisons using Mann–Whitney *U* test or Chi-square test, see Table 1 and, specifically for the SCR analysis, Supplemental Table 2 in the Supplemental Material.

**Table 1:** Participants’ demographics

	Guinea-Bissau (*n* = 33)	Portugal (*n* = 32)	Group comparison Guinea-Bissau vs. Portugal
Age (years; mean ± standard deviation)	25.9 ± 5.0	22.7 ± 5.2	*t*(63) = 2.47, p = .016*
Sex (male/female)	22/11	24/8	χ^2^(1) = 0.55, p = .460
University education (yes/no)	31/2	30/2	*p* > .999
Heterosexual (yes/no)	33/0	31/1	*p* = .492
Handedness (right/left)	32/1	32/0	*p* > .999
Drug addiction^a^ (yes/no)	0/33	1/31	*p* = .492
Drug use^b^ (yes/no)	0/33	8/24	*p* = .002*
Alcohol use^c^ (yes/no)	0/33	6/26	*p* = .011*
Tobacco use^d^ (yes/no)	1/32	1/31	*p* > .999
Mental health problems^e^ (yes/no)	6/27	3/29	*p* = .475
Family member with a mental illness^f^ (yes/no)	6/27	5/27	χ^2^(1) = 0.08, *p* = .783

Group comparisons between Guinea–Bissauan and Portuguese were performed with a two-sample *t* test for age and Chi-square test or the Fisher’s Exact Test (if there are less than 5 cases in one of the tested groups) for all the other variables and marked with an asterisk if significant at a level of *p* value < .05

^a^Personal history of drug addiction

^b^Use of recreational drugs in the last 6 months

^c^Consumes more than 28 units of alcohol per week (1 unit = ½ beer or 1 glass of wine)

^d^Smokes more than 5 cigarettes per day

^e^Participant had a diagnosed mental illness in the past

^f^Participant had at least one family member with a diagnosed mental illness

**Table 2:** Effect of nationality, emotion and ‘emotion x nationality’ on each behavioural and physiological measure

		Nationality (GB–PT)	Emotion	Emotion × nationality
Behavioural measures of emotion recognition	Accuracy	*F*(1, 60) = 56.40*,* FDR * p* < .001*, *η*_*p*_^*2*^ = .49, 95% CI [.30, .61], *d* = -1.82	*F*(5, 300) = 7.94*,* FDR * p* < .001*, *η*_*p*_^*2*^ = .12, 95% CI [.05, .17]	*F*(5, 300) = 16.58, FDR * p* < .001*, *η*_*p*_^*2*^ = .22, 95% CI [.13, .28]
	Response latency	*F*(1, 60) = 72.78, FDR * p* < .001*, *η*_*p*_^*2*^ = .55, 95% CI [.37, .66], *d* = 2.06	*F*(4.30, 257.71) = 18.73, FDR * p* < .001*, *η*_*p*_^*2*^ = .24, 95% CI [.14, .31]	*F*(4.30, 257.71) = 2.89, FDR *p* = .020*, *η*_*p*_^*2*^ = .05, 95% CI [.00, .09]
Behavioural measures of emotion authenticity	Rating	*F*(1, 62) = 0.87, FDR *p* = .355 *η*_*p*_^*2*^ = 0.01, 95% CI [.00, .11], *d* = -0.22	*F*(4.06, 251.55) = 18.64, FDR * p* < .001*, *η*_*p*_^*2*^ = .23, 95% CI [.13, .30]	*F*(4.06, 251.55) = 2.10, FDR *p* = .084, *η*_*p*_^*2*^ = .03, 95% CI [.00, .07]
	Response latency	*F*(1,62) = 14.24, FDR * p* < .001*, *η*_*p*_^*2*^ = .19, 95% CI [.04, .34], *d* = 0.90	*F*(2.68, 165.91) = 4.92*,* FDR *p* = .004*, *η*_*p*_^*2*^ = .07, 95% CI [.00, .13]	*F*(2.68, 163.20) = 2.63, FDR *p* = .084, *η*_*p*_^*2*^ = .04, 95% CI [.00, .10]
Physiological measures of emotion recognition (skin conductance response, SCR)	SCR amplitude	Wald χ^*2*^ (1, *N* = 211) = 3.39, *p* = .066, *d* = 0.24	Wald χ^*2*^ (5, *N* = 211) = 14.45, *p* = .013*	Wald χ^*2*^ (5, *N* = 211) = 2.83, *p* = .726
	SCR latency	Wald χ^*2*^ (1, *N* = 211) = 0.42, *p* = .519, *d* = 0.08	Wald χ^*2*^ (5, *N* = 211) = 23.43, *p* < .001*	Wald χ^*2*^ (5, *N* = 211) = 11.85, *p* = .037*
	SCR rise time	Wald χ^*2*^ (1, *N* = 211) = 0.09, *p* = .764, *d* = 0.04	Wald χ^*2*^ (5, *N* = 211) = 5.40, *p* = .369	Wald χ^*2*^ (5, *N* = 211) = 5.10, *p* = .403
	SCR percentage	Wald χ^*2*^ (1, *N* = 330) = 0.44, *p* = .506, *d* = -0.07	Wald χ^*2*^ (5, *N* = 211) = 2.84, *p* = .725	Wald χ^*2*^ (5, *N* = 211) = 5.89, *p* = .318

Effects statistically significant at a *p* < .05 are marked with an asterisk

### Power

Previous effect size reports a main effect of emotion (using the same task; *η*_*p*_^*2*^ = 0.54) (Lima et al. 2013), a main effect of nationality (*η*_*p*_^*2*^ = 0.76) (Gendron et al. 2014), and their interaction (*η*_*p*_^*2*^ = 0.21) (Gendron et al. 2014) on emotion recognition accuracy. This pointed, a priori, to a total needed sample size of 6, 8 and 14 subjects, respectively, for an 80% power and 5% alpha, calculated using G*Power 3.1.9.4 (Faul et al. 2009). The main effect of emotion on authenticity rating (*η*_*p*_^*2*^ = 0.70) (Lima et al. 2013) pointed to a total sample size of 4 subjects. (A post hoc sensitivity analysis is additionally available in Supplemental Material.) As our SCR analysis was unprecedented in this type of paradigm, reliable effect sizes to inform a power analysis were unavailable. Due to the exploratory nature of this analysis and that the sample was ~ 1/6 smaller than the behavioural analysis dataset, our SCR results should be interpreted as preliminary and suggestive, needing further replication in a larger sample.

### Emotion authenticity and emotion recognition in nonverbal vocalizations tasks

Each experimental session used the same equipment and took place in a similarly-sized, quiet, empty university classroom (at the Universidade de Lisboa in Portugal; or the Universidade Lusófona in Guinea-Bissau), and started with participants signing the informed consent and filling in a demographics questionnaire, which included questions targeting exclusion criteria. Participants were given earphones and the experimenters placed the electrodes for the recording of SCR (details regarding the SCR recording are in the ‘Skin conductance response recording and processing’ section). The volume of the computer task was kept the same and all participants were asked if they could hear the sounds well, during the training trials, to which they all replied affirmatively. Participants were instructed to complete the emotion authenticity task followed by the emotion recognition task (which included SCR recording). Each task employed the same set of 72 nonverbal emotional vocalization recordings of Portuguese origin (emotions: amusement, relief, pleasure, sadness, fear, anger; 12 items per emotion) we have previously recorded and validated (Lima et al. 2013) with a high recognition accuracy (average of 86% of correct classifications) (Lima et al. 2013). This set of stimuli was recorded from 4 actors and they were told not to produce sounds with verbal content. After reading the emotional label and a contextual sentence, they were asked to produce the sound they would when experiencing said emotion, as naturally and spontaneously as possible. The acoustic properties of amusement, pleasure, relief, anger, fear and sadness are, respectively: 982, 1257, 1034, 931, 876 and 1087 ms for duration; M = 73.3, SD = 9.7, M = 79.6, SD = 7.7, M = 69.6, SD = 10.2, M = 77.7, SD = 8.2, M = 72.0, SD = 13.0, M = 70.4, SD = 9.3 dB for intensity; and M = 327.4, SD = 133.4, M = 178.7, SD = 62.6, M = 468.5, SD = 135.6, M = 244.5, SD = 106.7, M = 420.1, SD = 63.1, M = 351.8 SD = 123.1 Hz for pitch. Participants were instructed to answer as quickly as possible. Before the testing phase of each task, six training-only items (each item lasts 10 s) were presented to the participants to check if the instructions were understood and for familiarization with both the task and the response keys. Both tasks were coded in PsychoPy v1.85.1 (Peirce 2009). Although participants were not asked specifically whether they could predict what was the native language or nationality of the vocalization encoder, we subjectively believe neither group could consciously and accurately ascertain this.

**Fig. 1:**
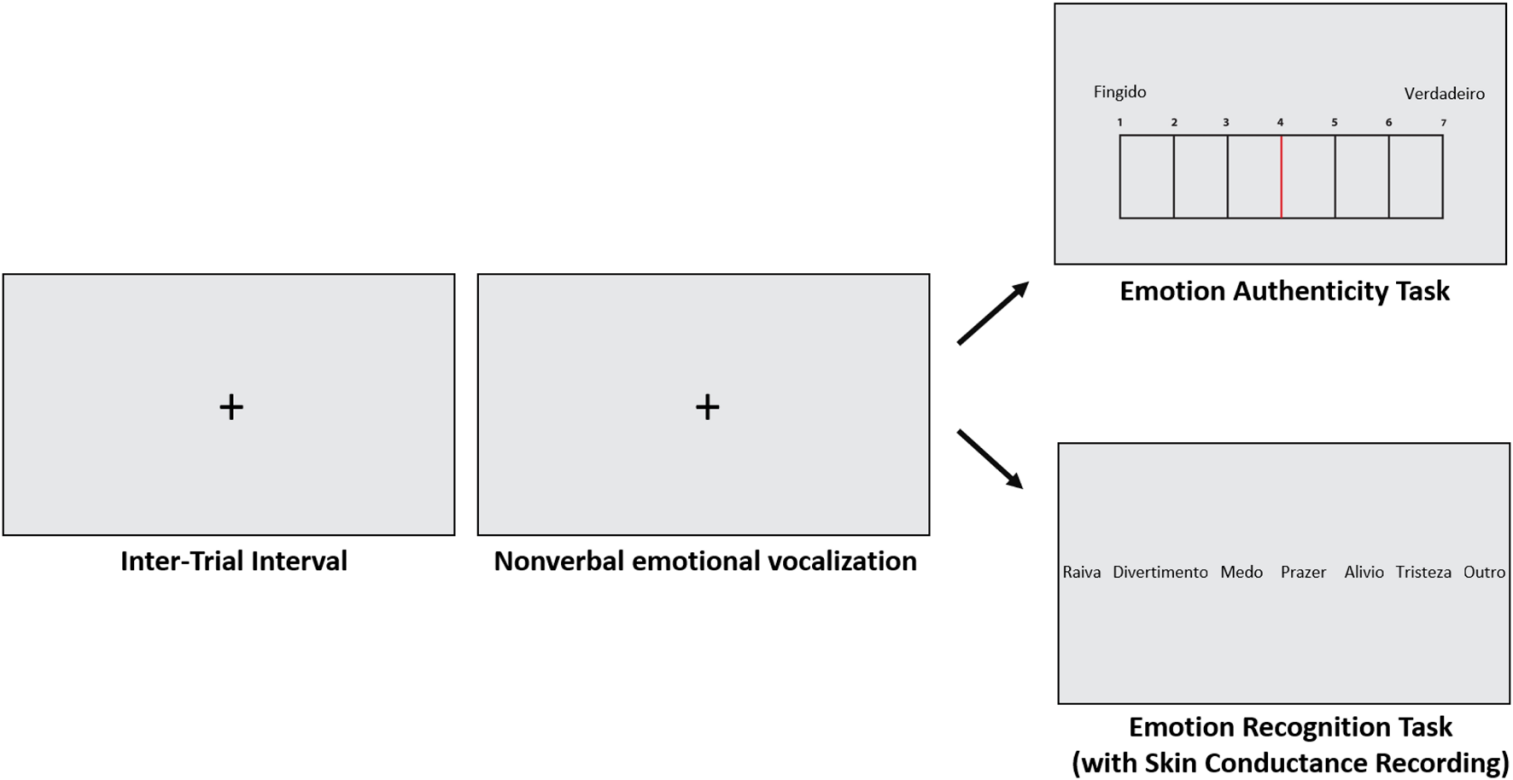
Presentation of the emotion authenticity and emotion recognition tasks. Both tasks had an inter-trial interval of 6 s followed by a nonverbal emotional vocalization presentation. Then, for the first task, participants rated the authenticity of the stimuli in a Likert scale 1–7, whereas for the second task, participants were asked to choose which of 6 emotions (or ‘other’) they recognized

In the emotion authenticity task, the participant heard the nonverbal emotional vocalizations, in a randomized order and with an inter-trial interval of 6 s, and had to rate the authenticity of the stimuli using a computerized Likert-type rating scale (1—completely fake, 7—completely genuine, chosen via the arrows of the keyboard and space bar) within a time limit of 6 s. The emotion recognition task used the exact same design and set of vocalizations as the emotion authenticity task, but instead of being asked to rate the authenticity of the stimuli, participants were instructed to identify the emotion conveyed in the nonverbal vocalization by selecting the appropriate emotion label displayed on-screen, as seen in Fig. 1, using the arrows of the keyboard and space bar. The participant could also select ‘other’, if they perceived that the displayed sound did not match any of the other possible answers. Here, the time limit for the participant response was set to 10 s after the end of the vocalization (Gendron et al. 2014).

Four response measures were then extracted for statistical analyses: (a) the rating and the response latency for the authenticity task; and (b) the accuracy and the response latency for the recognition task. The participant’s ratings were centred on level 4 of the rating scale (i.e. the neutral point of the 7-point rating scale) to make the interpretation more intuitive (i.e. a negative score correspond to a non-authentic vocalization; and a positive score correspond to an authentic vocalization), and were trial-averaged for each emotion level (i.e. amusement, relief, pleasure, sadness, fear, anger). Response latency for the authenticity task was computed as the average of all trials for each emotion level. Accuracy in the recognition task was calculated as the percentage of correct answers for all trials of each emotion level and response latency was computed as the average of all correct trials for each emotion level.

### Skin conductance response recording and processing

Skin conductance response (SCR) recording was performed using a Plugged Bitalino (da Silva et al. 2014) with a 10-bit channel of a sampling rate of 1000 Hz, during the recognition task. The performance of this equipment has been validated against that of other well-known instruments used for the same purpose (Batista et al. 2019; Batista et al. 2017). Two electrodes were placed on the palmar surface of the distal phalange of the second and third fingers of the non-dominant hand, as standard and resistant to room temperature differences (Measures 2012). The electrodes were self-adhesive with a 24 mm diameter, 1 mm of thickness, a core containing a polymer of coated Ag/AgCl and a conductive and adhesive hydrogel. Although room temperature was not controlled for, both data collection settings took place during similarly dry and warm weather months—spring (in Guinea-Bissau) and summer (in Portugal)—in similar non-artificially acclimatized rooms, with no occurrences of sweat or chills felt by participants. Electrodermal activity signals were monitored using OpenSignals (1.0.0.2) in a laptop connected to Plugged Bitalino via Bluetooth 2.0. Raw data were converted from OpenSignals to AcqKnowledge 4.2 (Biopac systems, Inc), using the guidelines from Bitalino specifications sheets for further processing. SCR recordings were then resampled to 250 Hz and a low pass filter of 2 Hz was applied. SCRs were detected automatically via AcqKnowledge routine and manually screened for artefacts and misdetections. SCR was scored as 1 or 0 for each trial using the following procedure: (a) the peak-to-peak amplitude difference in skin conductance of the largest response occurring in the 1–4 s temporal window after each vocalization onset was computed; (b) a minimal response criterion of 0.01 µS with a rejection threshold of 10% of the participant’s largest peak was used (Kim, Bang, & Kim, 2004); and (c) SCR was scored as 0 if they were below this criterion or 1 if above it. In addition, the latency, rise time and amplitude of the SCR for each trial were also measured. Furthermore, the amplitude of the SCR for each trial was additionally Lykken range-corrected following standard recommendations (Fowles et al. 2012; Lykken et al. 1966) and square root-transformed to meet parametric test assumptions (Rosa et al. 2017), to reduce error variance and increase statistical power when comparing different groups of participants.

Four measures were extracted for further statistical analyses: (a) percentage of SCR per emotion, computed as the sum of the SCR scores of all trials within each emotion level (i.e. amusement, relief, pleasure, sadness, fear, anger); and (b) the SCR latency, (c) rise time and (d) amplitude, by averaging the latency, rise time and amplitude, respectively, of the SCR of all correct trials (i.e. which participants were accurate in recognizing the emotion) by emotion level. In addition, for a complementary statistical analysis the SCR latency, rise time and latency were recomputed by averaging each one of them across all trials (i.e. irrespectively of recognizing correctly the emotion) by emotion level and reported in Supplemental Material. The basal SCL of each participant was obtained after resampling the signal to 250 Hz and low pass filtering of 2 Hz, and averaging it over a 30 s of a resting state condition just before the start of the emotion recognition task. One Guinea–Bissauan and nine Portuguese were excluded from SCR analysis due to technical problems with recording or absence of electrodermal response (which is consistent with the typical prevalence of hypo-responders of 10–25% in the population (Dawson et al. 2007)), resulting in a final sample of 32 and 23, respectively (Supplemental Table 2).

### Statistical analysis

Statistical analyses were conducted in SPSS (IBM Corp. Released 2017. IBM SPSS Statistics for Windows, Version 25.0. Armonk, NY: IBM Corp.). The main effects of emotion, nationality and their interaction on the behavioural measures were tested using two separate repeated measures Multivariate Analysis Of Variance (rpMANOVA) models: (a) one for the behavioural measures of the recognition task (accuracy and response latency), and (b) one for the behavioural measures of the authenticity task (rating and response latency). For the physiological analysis, the main effects of emotion, nationality and their interaction on SCR were estimated using one Generalized Estimating Equations (GEE) model per measure (percentage of SCR, SCR latency, rise time and amplitude), given its superior management of missing data (typically higher in SCR than behavioural data) in repeated measures designs, relative to ANOVA (Hubbard et al. 2010) (Fitzpatrick et al. 2019); this analysis was computed using only the correct trials to avoid the influence of performance differences on SCR and a chosen “Unstructured” covariance matrix between emotions. Both rpMANOVAs and the GEE were composed by two between-subject factors: nationality (Guinea-Bissau, Portugal), and sex (men, women; as a covariate of no interest, even though nationality groups were balanced for it) given its expected contribution to explaining the variance in behavioural and physiological measures (Lausen and Schacht 2018; Measures 2012); and one within-subject factor: emotion (amusement, relief, pleasure, sadness, fear, anger). Although age was not balanced between nationality groups, it was not included in the model as it did not show a statistically significant (*p* < 0.05) association with any of the behavioural or physiological measures. (Complementarily, the exploratory effect of emotion valence on the above-mentioned behavioural measures is reported in Supplemental Material.) In addition, to aid the interpretation of the above analyses, repeated measures correlations between accuracy and the other behavioural measures (perceived authenticity, and emotion recognition and authenticity latencies) were performed in R software 3.6 (R Core Team 2014) using the ‘rmcorr’ package (Bakdash and Marusich 2017). Furthermore, baseline skin conductance level (SCL) was compared between nationalities (Guinea-Bissau and Portugal) using a two-sample *t* test (and reported in detail in Supplemental Material).

Regarding the behavioural measures, for an overall testing of the null hypothesis, the rpMANOVAs were retrieved and reported in Supplemental Material. For null hypothesis testing of each specific behavioural measure, the corresponding univariate ANOVA was examined, with False Discovery rate (FDR) correction for the number of measures in each rpMANOVA (N = 2) (Benjamin and Hochberg 2009). For those ANOVA effects significant after FDR correction (*p* value < 0.05), follow-up post hoc pairwise comparisons between emotions were conducted and reported, again with FDR correction. As the ANOVA effect size measure, we used partial eta squared (*η*_*p*_^*2*^), and considered the following standard ranges: below 0.01 as marginal, 0.01–0.06 as small, 0.06–0.14 as medium, and above 0.14 as large effect sizes (Cohen 1977; Richardson 2011); in post hoc comparisons, we also report Cohen’s *d*. In addition, in the emotion recognition task, we tested whether Portuguese and Guinea–Bissauans matched the nonverbal vocalizations to their correct label at a level that exceeded chance by conducting two Chi-square tests, one for each nationality. Additional Chi-square tests were conducted for each specific emotion. We discussed effects, and considered them statistically significant, if showing a *p* value < 0.05, upon FDR correction. Unbiased hit rates, showing a similar pattern of “above chance-level” in similar degree for all emotions as the biased hit rates can be found as supplemental material (Supplemental Table 4).

Regarding physiological measures, null hypothesis testing results were retrieved from the GEE and reported in ‘Results’. We report and discuss all SCR results, regardless of multiple comparisons correction for the number of measures (*N* = 4) given that: (1) the SCR analysis was the first on vocalization’s emotion recognition to our knowledge, thus exploratory and preliminary; (2) mostly meant to aid interpretation of our behavioural data, and (3) the four SCR measures are highly inter-dependent. For the statistically significant effects, at *p* value < 0.05, follow-up post hoc pairwise comparisons between emotions were conducted and reported also regardless of correction. Herein, Cohen's *d* was calculated as the effect size using the GEE’s estimated marginal means.

## Results

### Emotion recognition task

#### Accuracy

Both Portuguese and Guinean-Bissauan listeners recognized West-European nonverbal vocalizations at a level that statistically significantly exceeded chance for each and all emotions [Portuguese: globally (χ^2^(30) = 7038.11, *p* < 0.001), and emotion-specifically (χ^2^(6) = 1744.25 for amusement, χ^2^(6) = 1408.22 for pleasure, χ^2^(6) = 1288.18 for relief, χ^2^(6) = 1096.63 for fear, χ^2^(6) = 1602.72 for anger and χ^2^(6) = 1006.04 for sadness; all *p* < 0.001); Guinea–Bissauan: globally (χ^2^(30) = 4230.43, *p* < 0.001), and emotion-specifically (χ^2^(6) = 1011.24 for amusement, χ^2^(6) = 291.21 for pleasure, χ^2^(6) = 718.99 for relief, χ^2^(6) = 865.73 for fear, χ^2^(6) = 1042.27 for anger and χ^2^(6) = 613.29 for sadness; all *p* < 0.001)].

**Fig. 2:**
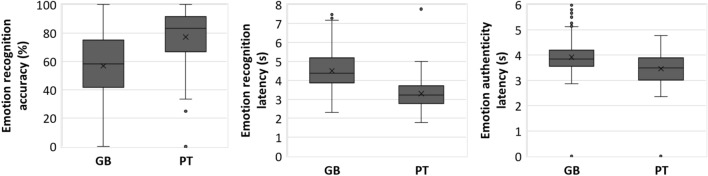
Main effects of nationality on accuracy (left) and response latency (middle) of the emotion recognition task, and on the response latency of the emotion authenticity task (right), with distribution (box plots) and mean values (cross) for each nationality (Guinea–Bissauan—GB, and Portuguese—PT). Only statistically significant main effects of nationality (at an FDR corrected *p* < .05) are shown

**Fig. 3:**
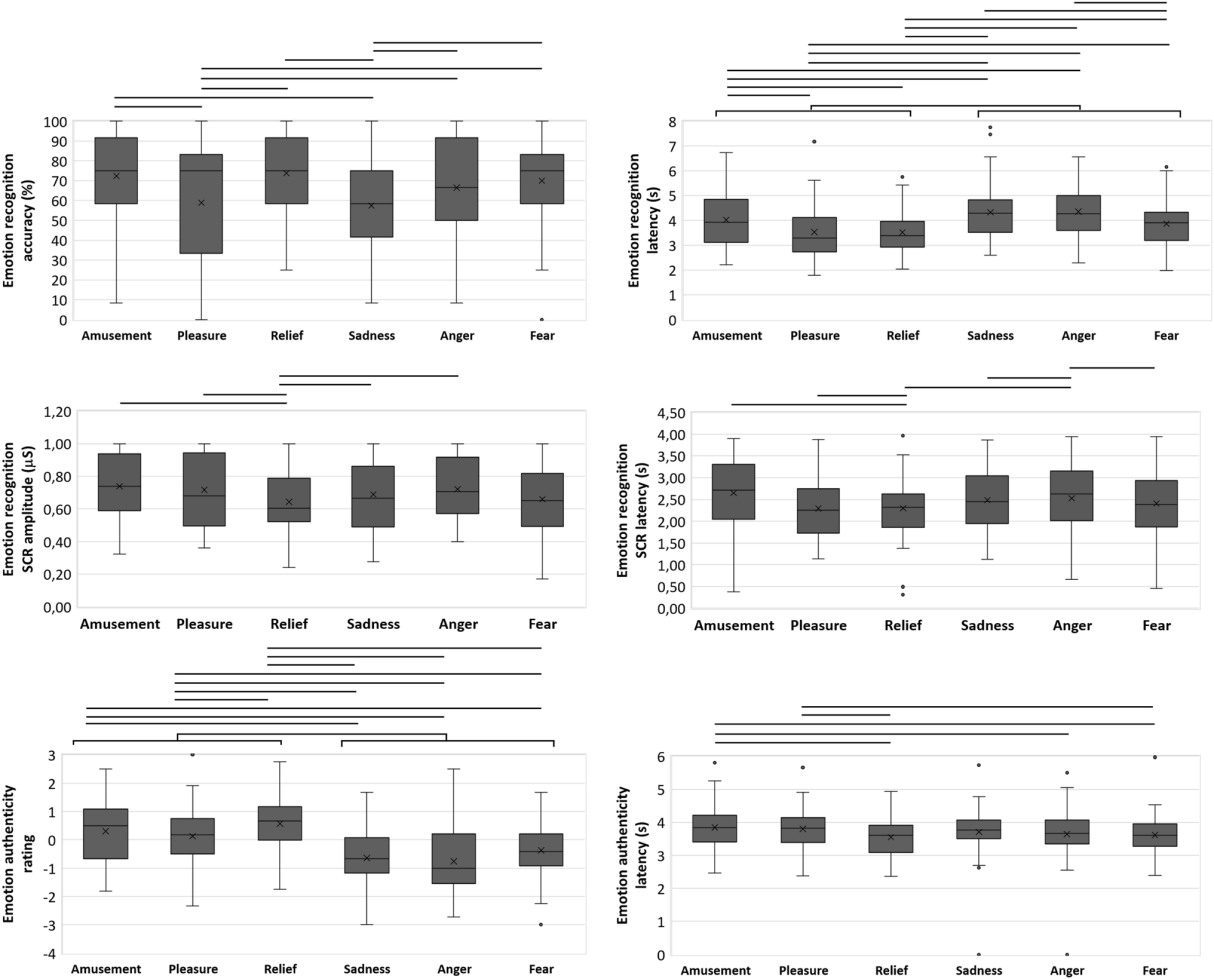
Main effects of emotion, on the accuracy (top left) and response latency (top right) of the emotion recognition task, on the rating (bottom left) and response latency (bottom right) of the authenticity recognition task, and on the SCR amplitude (middle left) and latency (middle right) during the emotion recognition task, with distribution (box plots) and mean values (cross) for each emotion (amusement, pleasure, relief, sadness, anger, and fear). Each line represents a statistically significant comparison (at an FDR corrected *p* < .05 for behavioural measures and at an uncorrected *p* < .05 for physiological measures) between emotions. Only statistically significant main effects of emotion (at an FDR corrected *p* < .05 for behavioural measures and at an uncorrected *p* < .05 for physiological measures) are shown

**Fig. 4:**
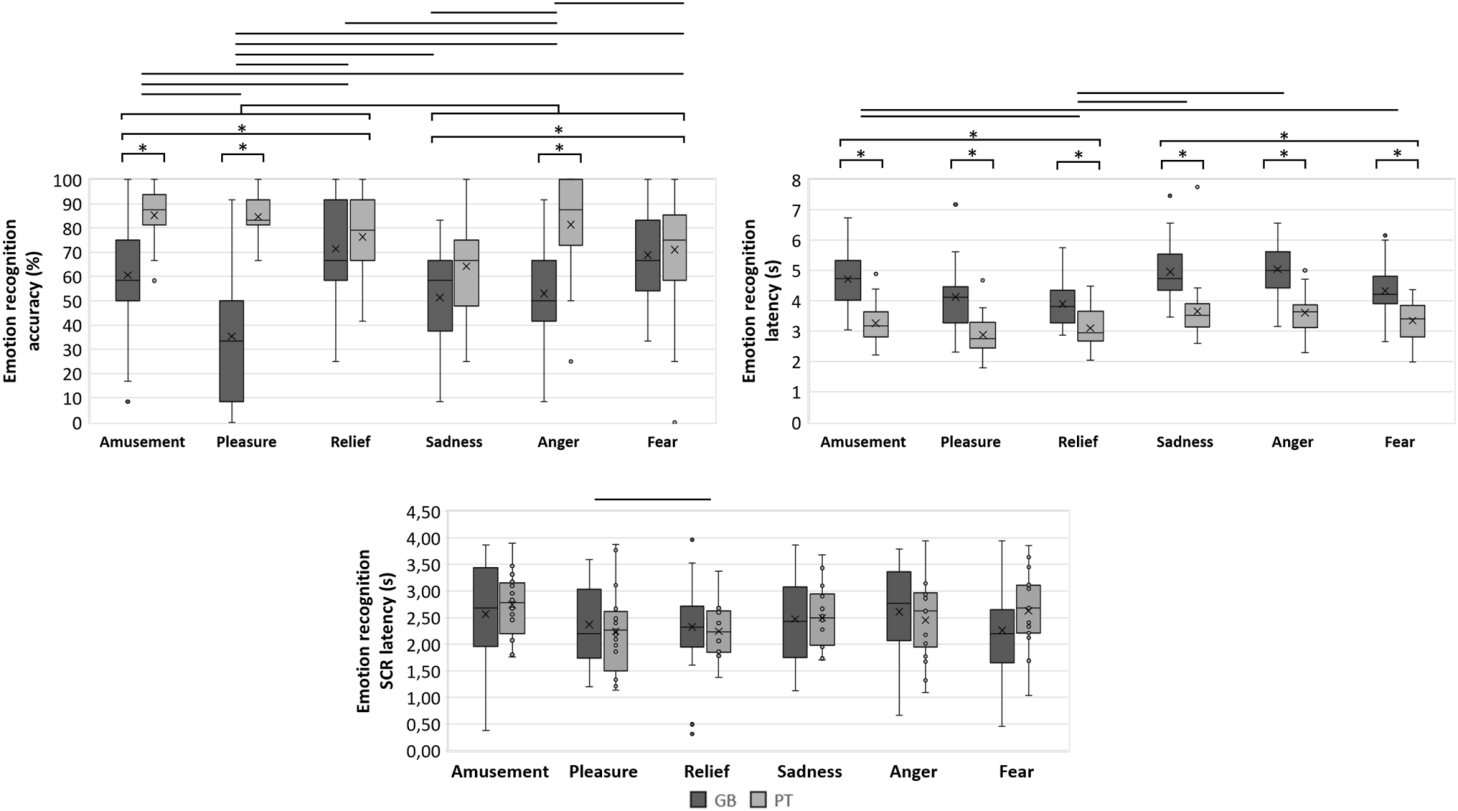
‘Emotion × nationality’ interaction effects on the accuracy (top left), response latency (top right) and concomitant SCR latency (bottom), for each emotion (amusement, pleasure, relief, sadness, anger, and fear) and nationality (Guinea–Bissauan and Portuguese), with distribution (box plots) and mean values (cross). Statistically significant post hoc comparisons (at an FDR corrected *p* < .05 for behavioural measures and at an uncorrected *p* < .05 for physiological measures) are marked as lines if between emotions and asterisks if between nationalities. Only statistically significant ‘emotion x nationality’ interaction effects (at an FDR corrected *p* < .05 for behavioural measures and at an uncorrected *p* < .05 for physiological measures) are shown

The main effect of nationality on emotion recognition accuracy was also statistically significant (*F*(1, 60) = 56.40, FDR corrected *p* < 0.001, *η*_*p*_^*2*^ = 0.49, 95% CI [0.30, 0.61], *d* = − 10.18, Table 2) with Guinea–Bissauan consistently performing at lower accuracy than Portuguese (Fig. 2). The main effect of emotion on recognition accuracy was statistically significant (*F*(5, 300) = 7.94, FDR corrected *p* < 0.001, *η*_*p*_^*2*^ = 0.12, 95% CI [0.05, 0.17], Table 2) and ordered from most to least recognizable: relief, amusement, fear, anger, pleasure and sadness (Fig. 3). Pairwise comparisons between emotions (Table 3 and Supplemental Table 1) showed significantly lower accuracies in recognizing: (1) pleasure versus amusement, relief, anger and fear; and (2) sadness versus amusement, relief, anger and fear (Fig. 3). The interaction of emotion by nationality was statistically significant (*F*(5, 300) = 16.58, FDR corrected *p* < 0.001, *η*_*p*_^*2*^ = 0.22, 95% CI [0.13, 0.28], Table 2) so that the effect of nationality was emotion-dependent when comparing pairwise: (1) between pleasure and each of all other emotions; (2) between amusement and relief or fear; and additionally (3) between anger and relief, sadness or fear (Fig. 4, Table 3 and Supplemental Table 1). That translated in Guinea–Bissauan being significantly worse than Portuguese in recognizing amusement, pleasure, sadness and anger (this difference being significantly larger in pleasure than in amusement, in sadness and in anger), but performing similarly in relief and fear.

**Table 3:** Post hoc pairwise comparisons for the main effects of emotion or ‘emotion x nationality’ interaction of Table 2 which were statistically significant on behavioural and physiological measures during emotion recognition and authenticity tasks (at *p* < .05 after FDR correction for multiple testing, except for the physiological measures)

Comparisons	Emotion recognition task														Emotion authenticity task			
	Accuracy				Response latency				SCR amplitude		SCR latency				Authenticity ratings		Response latency	
	Emotion		Emotion × nationality		Emotion		Emotion × nationality		Emotion		Emotion		Emotion × nationality		Emotion		Emotion	
	FDR *p*	*d*	FDR *p*	*d*	FDR *p*	*d*	FDR *p*	*d*	unc *p*	*d*	unc *p*	*d*	unc *p*	*d*	FDR *p*	*d*	FDR *p*	*d*
Amusement vs pleasure	.002*	0.46	.001*	–	< .001*	0.52	.554	–	.562	0.15	.785	0.06	.823	–	.411	0.11	.178	0.22
Amusement vs relief	.965	− 0.02	.002*		< .001*	0.60	.002*		.002*	0.76	.021	0.03	.214		.265	− 0.16	< .001*	0.87
Amusement vs sadness	< .001*	0.62	.051		.035*	− 0.28	.660		.169	0.30	.349	0.26	.726		< .001*	0.71	.110	0.25
Amusement vs anger	.228	0.17	.392		.007*	− 0.36	.919		.278	0.27	.168	− 0.28	.480		< .001*	0.55	.021*	0.35
Amusement vs fear	.228	0.17	.001*		.303	0.14	.033*		.056	0.42	.364	0.19	.568		< .001*	0.48	< .001*	0.82
Pleasure vs relief	.002*	− 0.47	< .001*		.766	− 0.04	.062		.012*	0.57	.028	0.43	.019*		.005*	− 0.38	< .001*	0.76
Pleasure vs sadness	.476	0.10	< .001*		< .001*	− 0.67	.894		.567	0.14	.313	0.20	.144		< .001*	0.60	.306	0.15
Pleasure vs anger	.023*	− 0.33	.003*		< .001*	− 0.84	.589		.590	0.11	.190	− 1.43	.761		< .001*	0.59	.219	0.20
Pleasure vs fear	.049*	− 0.29	< .001*		.004*	− 0.40	.344		.246	0.26	.524	0.13	.658		< .001*	0.52	< .001*	0.65
Relief vs sadness	.001*	0.54	.376		< .001*	− 0.81	.045*		.039*	− 0.40	.271	− 0.26	.913		< .001*	0.99	.297	− 0.17
Relief vs anger	.228	0.17	< .001*		< .001*	− 0.90	.009*		.022*	− 0.47	< .001*	− 0.74	.160		< .001*	0.77	.302	− 0.16
Relief vs fear	.228	0.17	.697		< .001*	− 0.54	.377		.214	− 0.27	.189	− 0.28	.223		< .001*	0.85	.474	− 0.10
Sadness vs anger	.001*	− 0.49	.003*		.766	− 0.04	.679		.829	− 0.04	.038	− 0.55	.504		.953	− 0.01	.873	0.02
Sadness vs fear	.002*	− 0.45	.139		.001*	0.47	.151		.544	0.12	.824	− 0.05	.610		.183	− 0.19	.423	0.12
Anger vs fear	.965	0.01	< .001*		.001*	0.48	.062		.421	0.16	.017	0.46	.136		.270	− 0.15	.474	0.10
Amusement _GB–PT_	−		< .001*	− 1.36	–		< .001*	1.89	–		–		.238	0.12	–		–	
Pleasure _GB–PT_			< .001*	− 2.15			< .001*	1.35					.189	− 0.15				
Relief _GB–PT_			.376	− 0.24			< .001*	1.15					.345	− 0.17				
Sadness _GB–PT_			.023*	− 0.60			< .001*	1.43					.624	0.17				
Anger _GB–PT_			< .001*	− 1.60			< .001*	1.76					.168	0.18				
Fear _GB–PT_			.719	− 0.09			< .001*	1.27					.204	0.07				

Statistically significant pairwise comparisons are marked with an asterisk [FDR *p* < .05 or uncorrected (unc) *p* < .05]

### Response latency

The main effect of nationality on the emotion recognition response latency was also statistically significant (*F*(1, 60) = 72.78, FDR corrected *p* < 0.001, *η*_*p*_^*2*^ = 0.55, 95% CI [0.37, 0.66], *d* = 11.55, Table 2), with Guinea–Bissauan showing a higher response latency than Portuguese (Fig. 2). The main effect of emotion on the response latency was significant (*F*(4.30, 257.71) = 18.73, FDR corrected *p* < 0.001, *η*_*p*_^*2*^ = 0.24, 95% CI [0.14, 0.31], Table 2) and ordered from highest to lowest latency: anger, sadness, amusement, fear, pleasure, and relief (Fig. 3). Pairwise comparisons of emotions (Table 3 and Supplemental Table 1) showed significantly faster responses in recognizing: (1) relief than amusement, sadness, anger, and fear; (2) fear than pleasure, sadness and anger; (3) pleasure than amusement, sadness, and anger; and (4) sadness and anger than amusement (Fig. 3). The interaction of emotion by nationality was statistically significant (*F*(4.30, 257.71) = 2.89, FDR corrected *p* = 0.020, *η*_*p*_^*2*^ = 0.05, 95% CI [0.00, 0.09], Table 2) so that the effect of nationality was different between relief and amusement, sadness, and anger and between amusement and fear. The effect was smaller in relief and fear, although Guinea–Bissauans were still significantly slower than Portuguese in recognizing all emotions.

## Skin conductance response during the emotion recognition task

### Skin conductance level at baseline

Baseline SCL was not statistically different between nationalities [t(53) = − 1.00, *p* = 0.324, M = − 3.81, SD = 2.81].

### Amplitude

There is a non-statistically significant main effect of nationality on SCR amplitude, albeit a trend was noted (Wald χ^*2*^ (1, *N* = 211) = 3.39, *p* = 0.066, d = 0.24, Table 2). The main effect of emotion on the SCR amplitude was statistically significant (Wald χ^*2*^ (5, *N* = 211) = 14.45, *p* = 0.013, Table 2) and ordered from the highest to the lowest amplitude: amusement, pleasure, anger, sadness, fear, and relief (Fig. 3). Pairwise comparisons of emotions (Table 3 and Supplemental Table 1) showed significantly lower SCR amplitudes in relief than amusement, pleasure, sadness, and anger (Fig. 3). The interaction of emotion by nationality was not significant (Table 2).

### Latency

The main effect of nationality was not significant (Table 2). The main effect of emotion on the latency of SCR was significant (Wald χ^*2*^ (5, *N* = 211) = 23.43, *p* < 0.001, Table 2) and ordered from the highest to the lowest latency: anger, amusement, pleasure, fear, sadness, and relief. Pairwise comparisons of emotions (Table 3 and Supplemental Table 1) show significantly lower SCR latency in relief than amusement, pleasure, and anger; and higher SCR latency in anger than sadness and fear (Fig. 3). The interaction of emotion by nationality was statistically significant (Wald χ^*2*^ (5, *N* = 211) = 11.85, *p* = 0.037, Table 2) such that the effect of nationality was different between pleasure and relief. The effect was smaller in relief, although Guinea–Bissauans showed lower SCR latency than Portuguese in both pleasure and relief.

### Rise time and SCR percentage

The main effects of nationality, emotion, and interaction emotion by nationality on the SCR rise time and percentage in the emotion recognition task did not reach statistical significance (Table 2).

## Emotion authenticity task

### Rating

The main effect of emotion on the rating was statistically significant (*F*(4.18, 254.70) = 17.02, FDR corrected *p* < 0.001, *η*_*p*_^*2*^ = 0.22, 95% CI [0.13, 0.29], Table 2) and ordered from most authentic to least: relief, amusement, pleasure, fear, sadness and anger (Fig. 3). Post hoc statistically significant (FDR corrected *p* < 0.05) pairwise comparisons between emotions (Table 3 and Supplemental Table 1) showed that (1) amusement was rated as more authentic than sadness, anger and fear; (2) relief was rated as more authentic than pleasure, sadness, anger and fear; and (3) pleasure was rated as more authentic than sadness, anger, and fear (Fig. 2). The main effect of nationality and the interaction of emotion by nationality were not significant (Table 2).

### Response latency

The main effect of emotion was significant (*F*(2.68, 163.20) = 4.80, FDR corrected *p* = 0.004, *η*_*p*_^*2*^ = 0.07, 95% CI [0.01, 0.15], Table 2) and ordered from highest to lowest response latency: amusement, pleasure, sadness, anger, fear and relief (Fig. 3). Pairwise comparisons of emotions (Table 3 and Supplemental Table 1) showed significantly higher response latency for (1) amusement than relief, anger and fear; and (2) pleasure than relief and fear (Fig. 3). The main effect of nationality (*F*(1, 61) = 14.80, FDR corrected *p* < 0.001, *η*_*p*_^*2*^ = 0.20, 95% CI [0.05, 0.36], *d* = 5.24) on the authenticity response latency was also significant (Table 2) with Guinea–Bissauan performing slower than Portuguese (Fig. 1) across emotions. The interaction emotion by nationality was not significant (**Table ****2**).

## Relationship between emotion recognition and authenticity tasks

The correlation between the behavioural measures from the emotional recognition task (accuracy and latency) is statistically significant such that they are negatively associated (*r*_*rm*_ (4039) = − 0.31, *p* < 0.001). The correlation between accuracy and authenticity rating is also significant such that they are positively associated (*r*_*rm*_ (4039) = 0.04, *p* = 0.004). Finally, the correlation between accuracy and latency in the authenticity task is not significant (*r*_*rm*_ (4039) = 0.00, *p* = 0.763).

## Discussion

In this study, we aimed to provide evidence of whether: (1) the universality hypothesis, and (2) an effect of nationality (as an aspect of culture), across or in dependence of emotion, both hold true using a context-free paradigm with nonverbal vocalizations, and upon controlling for the influence of socio-economic status, education, language, familiarity with the experimental setting. We compared Western European (Portuguese; in-group) and West African (Guinea–Bissauan; out-group) while exposed to Portuguese vocalizations in an emotion recognition task and an authenticity task using the same vocalizations set; using socio-economically, language and educationally matched samples. In regard to authenticity, we provide the first evidence that the perception of a socially complex attribute of nonverbal vocalizations may, like emotion recognition, be universal as well as differ between cultures and depend on the emotion. In addition, we replicate our own previous evidence of an influence of perceived authenticity on emotion recognition accuracy. In addition, for the first time in cross-cultural or emotion research, we add preliminary SCR data, to explore the peripheric nervous system correlates of emotion recognition and entice further work. In terms of predictions, besides expecting an above-chance level emotion recognition performance in support to the universality hypothesis, we also predicted a better performance—and a lower physiological response—from the Portuguese (as they were the decoders of the same culture as the stimuli’s encoders) than the Guinea–Bissauans in both tasks. As how this cultural effect would depend on the specific emotion, and how they would differ, we posed no a priori hypotheses, due to lack of previous evidence.

### Emotion recognition accuracy

The universality hypothesis was corroborated for all emotions, and both nationalities, as all were recognized above-chance level (only a small fraction of the responses was ‘other’, 8% on average, being relief the most predominant emotion to be answered ‘other’). Irrespective of nationality, we found different recognition accuracies between emotions, with emotion explaining 12% of the variance in accuracy otherwise left unexplained, i.e. left unexplained by nationality, the nationality by emotion interaction or sex. Ordered from most to least recognizable: relief, amusement, fear, anger, pleasure and sadness. Relief has been previously reported to be highly recognizable in nonverbal vocalizations (Schröder, 2003). However, relief has been equally recognized by educationally matched samples but differently recognized by those unmatched. (Cordaro et al. 2016). Consistent with previous literature, and irrespective of nationality, we observed: (1) pleasure to have lower recognizability compared to amusement (Lima et al. 2013; Sauter et al. 2010; Sauter and Scott 2007; Simon-Thomas et al. 2009), relief (Lima et al. 2013; Sauter et al. 2010; Sauter and Scott 2007; Simon-Thomas et al. 2009) and fear (Cordaro et al. 2016; Gendron et al. 2014); and (2) sadness to have the lowest recognizability compared to amusement (Lima et al. 2013; Sauter et al. 2010), relief (Sauter et al. 2010), anger (Laukka et al. 2013; Simon-Thomas et al. 2009) and fear (Laukka et al. 2013; Simon-Thomas et al. 2009). However, sadness has been found to be highly recognizable (F Johnson et al. 1986; Juslin and Laukka 2001; Thompson and Balkwill 2006), which is aligned with the proposition that it is a basic emotion (Paul Ekman 1999) highly relevant for survival (Elfenbein et al. 2007). Thus, our literature-conflicting results regarding sadness (Johnson et al. 1986; Juslin and Laukka 2001; Thompson and Balkwill 2006) may be due to vocalization production artefacts, that is, the untrained actors possibly relied on stereotypical portrayals of crying which might have influenced sadness recognition (Juslin and Laukka 2001). In the opposite extreme of this dimension, the basic emotion amusement (Paul Ekman 1999) being, with relief, the most well recognized emotion herein is coherent with literature (Gendron et al. 2014; Lima et al. 2013; Sauter et al. 2010; Sauter and Scott 2007). To portray amusement, the stimuli used here contained laughter which is a social cue observed in children as soon as 4 months old (Ruch and Ekman 2001), and in other species (Davila Ross et al. 2009), giving basis for its evolutionary roots.

Congruent with our hypothesis, we found a very large cultural effect on emotion recognition, given that nationality explained half (*η*_*p*_^*2*^ = 49%) of the variance in accuracy left otherwise unexplained (by the other model terms). Guinea–Bissauan were less accurate than Portuguese—which, in our study, is unlikely to be attributed to differences in education, language, socio-economic status or research setting. This is coherent with the literature, meta-analysis included (Elfenbein and Ambady 2002b; Gendron et al. 2014; Koeda et al. 2013; Sauter et al. 2010), and complements the only other education-controlled study (Cordaro et al. 2016) which also supports the universality hypothesis. Our results also point to a main effect of nationality in vocalizations’ emotion recognition. It is unclear whether this cultural effect represents an in-group advantage as this would only be testable with a balanced design (i.e. with the estimation of a ‘decoder’s nationality’ by ‘encoder’s nationality’ interaction, upon inclusion of Guinea–Bissauan vocalizations which we did not have available). In-group advantage has been shown in balanced studies individually, but also when unbalanced studies of both cultures/nationalities in questions were meta-analytically combined, showing similar (not statistically significantly different) effect sizes (Elfenbein and Ambady 2002a, 2002b; Matsumoto 2002). While it is still unclear why this advantage exists, possible remaining factors are cultural differences in emotion expression (Scherer et al. 2001) and/or in emotional concepts (Russell 1994), for example.

The interaction between emotion and nationality had a large effect, explaining 22% of the variance in emotion recognition accuracy left otherwise unexplained (i.e. by each factor individually or sex), and showing that the nationalities were significantly different (pairwise): (1) between pleasure and each of all other emotions; (2) between amusement and relief or fear; and additionally (3) between anger and relief, sadness or fear. In sum, the Portuguese were more accurate while recognizing: (1) amusement, pleasure and anger (this difference being significantly larger in pleasure than in amusement, and also than in anger), but not relief, sadness and fear. As alluded to above, this cultural effect of consistently superior performance by the Portuguese could be due to the shared cultural origin with the vocalization expressors, which might be underlined by the presence of an in-group advantage, warranting future testing with a balanced design study. It is possible culture influences emotion expression and recognition which bias individuals to highlight or reject specific emotional features, according to some socially acquired norms. These norms are shared between members of the same group, thus helping them communicate, whereas out-group members are oblivious to them (Elfenbein and Ambady 2002b). Therefore, it is expected that some emotions are more influenced by cultural modulation than others, based on their importance in being communicated within or across groups. It makes sense that for emotions that are used to strengthen bonds between group members (Shiota et al. 2004) (such as for positive and negative reinforcement of in-group member’s behaviour) would be more affected by culture, and thus present an in-group advantage, while emotions that are used to communicate with out-group members would be more culture-independent. Others have usually aligned this division with emotional valence (Elfenbein and Ambady 2002b; Koeda et al. 2013; Sauter et al. 2010), where positive emotions are hypothesized to be more prone to in-group advantage than negative ones, however, this is likely to be an oversimplification (Jürgens et al. 2013). In fact, other criteria for grouping emotions may be more useful for this purpose (Simon-Thomas et al. 2009), for example, amusement and relief may be considered to be in the ‘epistemological’ family (i.e. emotions that accompany changes one understands about the environment); and pleasure in the ‘savouring’ family (i.e. emotions that involves introspective experiencing) (Simon-Thomas et al. 2009). Another oversimplification to be considered is that cultural specificity may depend on modality of expression: i.e. amusement showed larger in-group advantage for vocalizations than for facial expressions (Elfenbein and Ambady 2002b) while the reversed occurred in anger and sadness; fear resulted in a large in-group advantage in both modalities whereas anger resulted in a small in-group advantage in both.

Our results show the Portuguese were better at recognizing amusement than Guinea–Bissauan, an emotion which is thought to be socially impactful (Vettin and Todt 2005), but contested by previous nonverbal vocalization studies in which cultural advantage was not observed (Gendron et al. 2014; Sauter et al. 2010). We also found Portuguese were better at recognizing pleasure (in fact the emotion where we found the largest difference between both nationalities) which is supported by one study (Sauter et al. 2010) and hinted by another (Gendron et al. 2014). For relief, our results do not seem to corroborate previous findings that show cultural differences in its recognition (Gendron et al. 2014; Laukka et al. 2013; Sauter et al. 2010). Regarding fear and sadness, Guinea–Bissauan were as good as Portuguese to recognize them, consistent with previous work (Sauter et al. 2010), including a meta-analysis (Elfenbein and Ambady 2002b). As the perception of fear in others may signal an imminent threat (Skuse 2003) critical for our survival, is it plausible that this ability is so biologically hard-wired that it overcomes the subtle effects of cultural specificity. This would also explain our similar finding for sadness, which in our study is expressed as crying, and as such also signals imminent threat or hazard—besides being, in nature, majorly expressed by children which do not have the opportunity to learn in-group social norms. For anger, we observed a clearly better performance of the Portuguese in its recognition, suggestively contesting previous work (Sauter et al. 2010). Interpretation of this remains unclear as, given its importance for threat detection, no difference between nationalities might be expected instead (Grandjean et al. 2005; Sander et al. 2005). In sum, our results support, with no influence from factors such as language, social, economic and educational status, the universality hypothesis, and an effect of nationality (potentially reflecting an underlining in-group advantage to be further tested in a balanced design study). Our methodological innovation strongly supports and validates these constructs while also extending their generalizability by testing two cultures under-studied in cross-cultural emotion recognition.

### Emotion recognition response latency

Emotion explained 24% of the variance in response latency which was otherwise left unexplained by the other model terms. Emotions were ordered from highest to lowest latency as in: anger, sadness, amusement, fear, pleasure, relief. Irrespective of nationality, subjects were faster in recognizing: (1) relief than amusement, sadness, anger, and fear; (2) fear than sadness and anger; and (3) pleasure than amusement, sadness, and anger. Having two negative valenced stimuli with highest response latency may be contrary to the survival-relevant signal processing evolutionary expectation that they should be recognized faster, but research has shown that when participants are required to categorize an emotional stimulus (e.g. expressions and words), responses to negative valenced tend to be slower than positive valenced stimuli (Ducci 1981; Eastwood et al. 2003; Hugenberg 2005; Kirita and Endo 1995; Leppänen et al. 2003; Stenberg et al. 1998). Guinea–Bissauan were slower to respond than Portuguese, irrespective of emotion, with nationality explaining more than half (*η*_*p*_^*2*^ = 55%) of the variance in accuracy left otherwise unexplained (by emotion, the 2-way interaction or sex); which can be interpreted as an indicator of higher difficulty and thus, cognitive processing load by the Guinea–Bissauan (Leppänen and Hietanen 2004). In addition, the dependence of the effect of nationality on emotion explains 5% of the variance in response latency left unexplained by the other factors individually, although Guinea–Bissauans were still significantly slower than Portuguese in recognizing all emotions.

Considering the congruency of the main effect of emotion in accuracy and response latency, relief was the easiest emotion to recognize (i.e. recognized the fastest and most accurately). In contrast, sadness was the hardest to recognize and second slowest, compared to most emotions. For amusement, accuracy and response latency increase in the same direction, suggesting participants required more time to process the vocalization to judge it correctly. The opposite was observed for pleasure where participants had difficulties in recognizing the emotion (ranks second last in accuracy), but once correctly classified, they responded quickly (ranks second last in response latency). Regarding anger and fear, their degree of accuracy ranks similarly to their degree of response latency for each respective emotion (and generally lower than positive emotions). For the main effect of nationality, the results are consistent with our hypothesis where Guinea–Bissauan participants were less accurate and slower than the Portuguese, in each and every emotion. Considering the interaction emotion by nationality on accuracy and response latency, relief and fear stand out: the Guinea–Bissauan showed increased difficulty (response latency) while reaching similar performance (accuracy) in this dimension, compared to Portuguese. For all other emotions, Guinea–Bissauans took longer to respond and still performed worse than the Portuguese, demonstrating a clear disadvantage in recognizing Western European vocalizations.

## Skin conductance during emotion recognition

Our hypothesis of higher arousal or cognitive information processing load while recognizing cross-cultural emotional vocalizations, and our above-discussed finding of lower performance in cross-cultural recognition, is congruent with the trend (*p* = 0.066) we found towards Guinea–Bissauan showing, irrespective of emotion, a higher SCR amplitude than Portuguese. Altogether, this suggests higher autonomic arousal may be deriving from higher cognitive effort (coming from higher perceived task demand).

To our knowledge, we also provide the first report, for nonverbal vocalizations, that SCR amplitude, as well as latency, significantly (*p* < 0.05) differ depending on the emotion concerned. In our data, both measures follow the same pattern, being that those showing higher amplitude also showed higher latency: (1) amplitude was lower for relief compared to amusement, pleasure, sadness and anger, and higher for anger compared to sadness and fear; (2) latency was lower for relief compared to amusement, pleasure, and anger; and higher for anger compared to sadness and fear. In regard to latency, nonverbal vocalization findings, such as ours, seem thus not to easily comparable as those of prosodic vocalizations given that sadness and anger prosody has shown higher SCR amplitudes compared to amusement (happy) (Petrone et al. 2016)—which we do not find significantly different pairwise. In addition, discrepantly with our findings, that study found lower SCR latencies for prosodic anger compared to neutral, amusement (happy) and sadness.

Whilst SCR amplitude is robustly associated to event-related cognitive load arousal (MacPherson et al. 2017; Nourbakhsh et al. 2017), latency is less well characterized during event-related paradigms. Nevertheless, accepting as analogy the autonomic system’s pupil response wherein both amplitude/peak (van der Wel and van Steenbergen 2018) and latency (Kahneman and Beatty 1966) are positively associated to cognitive load, and pupil positively associated with SCR (Wang et al. 2018), it is plausible that *both* SCR measures are/become higher when events are more cognitively demanding. This might explain the congruency we found between our SCR amplitude and latency between different emotions and point to the suggestion that these differences reflected different degrees of difficulty in identifying the emotions.

Regarding how emotion may impact on the nationality effects, we found a statistically significant interaction effect of nationality by emotion, such that in pleasure, Guinea–Bissauans showed lower SCR latency than Portuguese, and in relief, the opposite. Nevertheless, the difference between nationalities is not significant in any of the emotions individually, which makes any interpretation of this interaction speculative. In sum, we consider our SCR results exploratory, and that a more conclusive interpretation would warrant further independent findings using a similar paradigm.

### Emotion authenticity rating

In the perceived authenticity task, emotion explained 22% of the variance in rating, irrespective of nationality (ordered from most authentic to least: relief, amusement, pleasure, fear, sadness and anger). We found that: (1) amusement was rated as more authentic than sadness, anger and fear; (2) relief was rated as more authentic than pleasure, sadness, anger and fear; (3) pleasure was rated as more authentic than sadness and anger; (4) and fear was rated as more authentic than sadness and anger. Finally, we also found that as a group, positive emotional vocalizations (amusement, pleasure, relief) were rated as more authentic than negative ones (sadness, anger, fear). This replicates what we have reported earlier using the same stimuli library with another Portuguese sample (Lima et al. 2013). It is unclear why such a difference exists, but a possible explanation is that authentic negative emotions are more difficult to portray, therefore, participants rate negative vocalizations as being less authentic. Neither main nor interaction effects of nationality on the ratings reached statistical significance (discussed below).

### Emotion authenticity response latency

Regarding perceived authenticity response latency, emotion explains 6% of the variance in response latency, irrespective of nationality (ordered from highest to lowest latency: amusement, pleasure, sadness, anger, fear and relief). Concretely, we found higher latencies for amusement and pleasure, each compared to relief and fear. Nationality showed, again, a large main effect explaining 20% of the variance in latency unexplained by emotion. The increased response latencies by Guinea–Bissauan participants indicate they encountered more difficulty when making authenticity judgements, which was also seen for the emotion recognition task. This may seem surprising given the null effect on the rating. Yet, that null effect is corroborated by previous results (Jürgens et al. 2013) of multiple cultures (dichotomously) rating authenticity poorly between themselves. For future research, it would be advisable to manipulate vocalizations authenticity to better pinpoint the effect that this variable may have on the recognition of emotions across cultures.

### Relationship between tasks and behavioural measures

Not surprisingly, we found, for the emotion recognition task, that accuracy was negatively correlated to response latency (i.e. the harder subjects found the task the longer they took to respond). In addition, this emotion recognition accuracy improves with the perceived authenticity rating, in line with our previous results (Lima et al. 2013), but not with the time subjects took to decide on the authenticity rating.

### Potential limitations

First, skin colour may result in differences in SCR given that black skins have shown lower basal SCL (Boucsein 2012; Johnson and Landon 1965), and lower electrodermal reactivity to general tones and noises (Boucsein 2012) compared to whites, possibly due to the lower density of sweat glands and thicker outermost skin layer (Boucsein et al. 2012; Johnson and Landon 1965; Juniper and Dykman 1967). However, although all our Guinean-Bissauans were Black and all our Portuguese participants White, given that we found no differences in basal SCL at baseline (prior to task), and that our SCR findings pointed to a trend towards higher (rather than lower) SCR amplitude in Guinea–Bissauan compared to Portuguese, such prior effects could not have posed as confounding factors in our SCR analysis (Bernstein 1965; Janes et al. 1978; Johnson and Landon 1965). Moreover, we minimized between-subjects variability by applying a standard range correction—Lykken correction (whereby SCRs are divided by the individual’s maximal SCR), reducing error variance and thus increasing statistical power in-group comparisons. Second, since both nationality groups spoke the same language at university level, we were not hindered by reliance on translation of the discrete words to be matched, yet different connotations in each culture might exist, which may have not have been avoided. Third, as we have not administered tasks to our participants other than the emotional ones reported, we could not ascertain, regarding response latency specifically, whether Guinean-Bissauans were slower that Portuguese in emotion recognition and emotion authenticity specifically, or in psychological tasks in general. Nevertheless, we would not have a reason to suspect motricity differences between Guinea–Bissauans and Portuguese, and note that both participant groups speak the same language, were balanced in terms of social, economic and educational strata, interacted daily with computers, had a 18–45 years of age range and were healthy. Forth, we note that, as common in tasks with emotional stimuli (Gendron et al. 2014; Laukka et al. 2013; Lima et al. 2013; Sauter et al. 2010) the stimuli used were acted, and thus generalization of findings to genuine vocalizations is obviously limited. Nevertheless, the accuracy of subjects of both nationalities was above-chance level, in this and previous studies employing the same acted stimuli set (Lima et al. 2013)—suggesting the stimuli are convincing. Moreover, this would be a minor concern given that our main interest was the comparison between nationalities in terms of their relative quantitative accuracy, and their above-chance level accuracy.

Fifth, although there is a lively debate on the validity of different methods to test in-group advantages (Elfenbein and Ambady 2002a, 2002b; Matsumoto 2002), there is incontestable superiority of the ‘balanced design’ to detect in-group advantages, as it allows a ‘decoders’ nationality’ by ‘encoders’ nationality’ interaction to be estimated. If both cultures show performance superiority towards their in-group stimuli, in a “double cross cultural” study then an in-group advantage can be ascertained given that no extraneous confounding factors (such as cognitive skills, stress, unfamiliarity with the research setting or language familiarity—which could be different between the cultural decoder groups) could be explaining the superior performance of both in-group decoders. We note that we have not used such a balanced comparison design, and therefore, we cannot ascertain that our main effect of cultural group is underlined by an in-group advantage effect. However, we have used a balanced sampling design, meaning that we have protected our analysis from potential confounders making a potential in-group advantage of the Portuguese effect possible. Our subjects were either university students or recently graduated, of the biomedical sciences field taught in Portuguese, using Portuguese as a primary language, and commonly using laptop computers. Subjects were also tested in a room at their own (familiar) university campi, and were equally unfamiliar with psychophysiological or psychological experiments. Further research is warranted using a balanced design for the vocalized emotions’ recognition of Portuguese and Guinea–Bissauan to complement the present study’s contribution.

Lastly, regarding our test for the universality hypothesis, we understand there is debate on whether the “above-chance level” criteria is the best to infer universality, because the valence of the stimuli may confound the recognition of discrete emotions, resulting in inflated recognition rates (i.e. recognition rates could be low but still above what is expected by chance if participants are able to distinguish positive from negative expressions) (Cordaro et al. 2016; Gendron et al. 2014; Russell 1994). However, the vast majority of the emotion recognition studies (all except one (Cordaro et al. 2016), as far as we know) consider that the test against chance level is sufficient to indicate that the participants correctly perceived the emotional construct (Elfenbein and Ambady 2002b; Gendron et al. 2014; Jürgens et al. 2013; Laukka et al. 2013; Lima et al. 2013; Sauter et al. 2010), and agreeing with this view, we followed the norm. Furthermore, we note that in our study, the overall recognition rate was 68.7%, i.e. more than four times what would be expected by chance (i.e. for 6 emotions: 16.6%) which lends large support for the universality hypothesis, and is on par with other cross-cultural emotion recognition studies (Laukka et al. 2013).

### Conclusion

In summary, when testing Western Europeans and Western Africans’ recognition of Western European nonverbal emotional vocalizations, we found that although both groups recognized all emotions above-chance level, in line with the universality hypothesis, there were significant main effects of emotion and of nationality, and of their interaction. Emotion recognition was more accurate and faster, across emotions by the Portuguese (in-group, i.e. which had the same nationality as the vocalizations encoders) than Guinea–Bissauans, particularly in pleasure, amusement and anger. This reinforces some cultural specificity in emotion recognition by which culture modulates emotion expression and recognition, strengthening communication of emotions within cultures. Congruently, Portuguese showed a trend for a lower autonomic sympathetic system response (in skin conductance amplitude) than Guinea–Bissauan, and a suggestively different SCR latency between relief and pleasure. We have not found evidence that culture impacted on perceived authenticity, in the same emotions, albeit in-group participants were faster to respond. In conclusion, our evidence suggests that emotion recognition, even at the level of nonverbal emotional vocalizations, can be subtly modulated by culture, even when controlling for socio-economic-educational and language differences. In addition, we also provide an unprecedented, and thus preliminary, account of how differently six emotions, expressed via nonverbal vocalizations, elicit an autonomic sympathetic system response measured with skin conductance.

## Supplementary Information

Below is the link to the electronic supplementary material.

Supplementary file1 (DOCX 125 KB)

## Data Availability

The data collected for this work is publicly available at: https://doi.org/10.7910/DVN/IQHNCB.

## Declarations

### Public significance statements

Emotion recognition in nonverbal vocalizations shows a cultural/national/ethnic advantage even for similar socio-economic-educational status and language. Emotion-wise, this cultural advantage may exist for pleasure, amusement, and anger, but less for sadness, relief and fear. Preliminary skin conductance response evidence suggests this cultural advantage reflects a lower arousal or cognitive load, across emotions. The skin conductance response differences elicited by 6 nonverbally vocalized emotions is also described, for the first time.
