# Supplementary material for: Cultural differences in vocal emotion recognition: a behavioural and skin conductance study in Portugal and Guinea-Bissau
Source: Psychol Res. 2021 Mar 15;86(2):597–616. doi: 10.1007/s00426-021-01498-2 (PMC8885546; doi:10.1007/s00426-021-01498-2)
Supplement: Supplementary file 1 — Supplementary file1 (DOCX 125 KB) [file 426_2021_1498_MOESM1_ESM.docx]

**Cultural differences in vocal emotion recognition: A behavioural and skin conductance study in Portugal and Guinea-Bissau**

Gonçalo Cosme1*, MSc, Vânia Tavares1,2*, MSc, Guilherme Nobre2, BSc, César Lima3, PhD, Rui Sá4,5, PhD, Pedro Rosa3,6, PhD, Diana Prata1,3,7, PhD

**Supplemental Material**

[**Post hoc sensitivity power analysis** 3](#_Toc59355643)

[**1. Emotion recognition task** 3](#_Toc59355644)

[***Accuracy*** 3](#_Toc59355645)

[***Response Latency*** 3](#_Toc59355646)

[**2. Emotion authenticity task** 4](#_Toc59355647)

[***Ratings*** 4](#_Toc59355648)

[***Response Latency*** 4](#_Toc59355649)

[**Behavioural repeated measures MANOVA overall effects** 5](#_Toc59355650)

[**1. Emotion recognition task** 5](#_Toc59355651)

[**2. Emotion authenticity task** 5](#_Toc59355652)

[**‘Nationality by Valence’ Interactions** 6](#_Toc59355653)

[**1. Emotion recognition task** 6](#_Toc59355654)

[***Accuracy*** 6](#_Toc59355655)

[***Response latency*** 6](#_Toc59355656)

[**2. Emotion authenticity task** 6](#_Toc59355657)

[***Rating*** 6](#_Toc59355658)

[***Response latency*** 7](#_Toc59355659)

[**Skin conductance response across all trials during the emotion recognition task** 8](#_Toc59355660)

[**1. Amplitude** 8](#_Toc59355661)

[**2. Latency** 8](#_Toc59355662)

[**3. Rise time** 8](#_Toc59355663)

[**4. Percentage** 9](#_Toc59355664)

[**Confusion patterns and unbiased hit rates** 9](#_Toc59355665)

[**1. Emotion recognition task** 9](#_Toc59355666)

# **Post hoc sensitivity power analysis**

The sensitivity power analysis was conducted using the G*Power 3.1.9.4 software for the following effects: main effect of emotion (Test family: F tests; Statistical test: ANOVA: Repeated measures, within factors); main effect of nationality (Test family: F tests; Statistical test: ANOVA: Repeated measures, between factors); and interaction effect of emotion by nationality (Test family: F tests; Statistical test: ANOVA: Repeated measures, within-between interaction). For all power analysis tests, significance level (α err prob), power (1-β err prob), Number of groups, and Number of measurements were set to 5%, 80%, 2 (Guinea-Bissau and Portugal), and 6 (amusement, relief, pleasure, sadness, fear, anger), respectively. Additionally, effect size specification was set ‘as in SPSS’. Measure-specific G*Power input parameters are described below.

## **1. Emotion recognition task**

### ***Accuracy***

Additional G*Power input parameters: Total sample size = 63 subjects; Nonsphericity correction ε = 1. The smallest possible effect size (*η_p_^2^*) that can be detected with 63 subjects is *η_p_^2^* = .04 for the main effect of emotion, *η_p_^2^* = .12 for the main effect of nationality, and *η_p_^2^* = .04 for the interaction effect of emotion by nationality on the emotion recognition accuracy.

### ***Response Latency***

Additional G*Power input parameters: Total sample size = 63 subjects; Nonsphericity correction ε = 0.859. The smallest possible effect size (*η_p_^2^*) that can be detected with 63 subjects is *η_p_^2^* = .05 for the main effect of emotion, *η_p_^2^* = .12 for the main effect of nationality, and *η_p_^2^* = .05 for the interaction effect of emotion by nationality on the response latency.

## **2. Emotion authenticity task**

### ***Ratings***

Additional G*Power input parameters: Total sample size = 64 subjects; Nonsphericity correction ε = 0.835. The smallest possible effect size (*η_p_^2^*) that can be detected with 64 subjects is *η_p_^2^* = .05 for the main effect of emotion, *η_p_^2^* = .12 for the main effect of nationality, and *η_p_^2^* = .05 for the interaction effect of emotion by nationality on the emotion recognition ratings.

### ***Response Latency***

Additional G*Power input parameters: Total sample size = 64 subjects; Nonsphericity correction ε = 0.535. The smallest possible effect size (*η_p_^2^*) that can be detected with 64 subjects is *η_p_^2^* = .06 for the main effect of emotion, *η_p_^2^* = .12 for the main effect of nationality, and *η_p_^2^* = .06 for the interaction effect of emotion by nationality on the response latency.

# **Behavioural repeated measures MANOVA overall effects**

## **1. Emotion recognition task**

Overall (from repeated measures MANOVA), there was a statistically significant main effect of emotion (Pillai’s Trace *V* = .70, *F*(10,51) = 11.97, *p* < .001, *η_p_^2^* = 0.70), nationality (Pillai’s Trace *V* = .60, *F*(2,59) = 45.00*, p* < .001, *η_p_^2^* = 0.60), and ‘emotion x nationality’ (Pillai’s Trace *V* = .60*,* *F*(10,51) = 7.72, *p* < .001, *η_p_^2^* = 0.60) on the behavioural measures of the emotion recognition task. [Greenhouse-Geiser correction to the degrees of freedom was applied to the univariate ANOVAs of the response latency, given that Mauchly’s test of sphericity indicated that the assumption of sphericity had been violated for response latency (*χ*^2^(14) = 24.43, *p* = .041), albeit not for accuracy (*χ*^2^(14) =11.32, *p* = .661).]

## **2. Emotion authenticity task**

Overall there was a statistically significant effect of emotion (Pillai’s Trace *V* = .78, *F*(10,53) = 18.58, *p* < .001 *η_p_^2^* = .79), nationality (Pillai’s Trace *V* = .19, *F*(2,61) = 7.19*, p* = .002, *η_p_^2^* = 0.19), and ‘emotion x nationality’ interaction (Pillai’s Trace *V* = .43, *F*(10,53) = 3.97, *p* < .001, *η_p_^2^* = 0.43) on the behavioural measures of the authenticity task. [A Greenhouse-Geiser correction to the degrees of freedom was applied to the univariate ANOVAs, given that Mauchly’s test of sphericity indicated that the assumption of sphericity had been violated for both the authenticity rating (*χ*^2^(14) = 36.79, *p* = .001) and the response latency (*χ*^2^(14) = 141.01, *p* < .001)].

# **‘Nationality by Valence’ Interactions**

## **1. Emotion recognition task**

### ***Accuracy***

The pairwise comparison of emotion valence was not statistically significant (**Supplemental Table 1**).

The interaction of emotion valence by nationality was statistically significant [*F*(1,60) = 8.85, *p* = .004, *η_p_^2^* = 0.12, 95% CI [0.01 0.29], **Supplemental Table 1**] so that the Guinea-Bissauan performed significantly worse than the Portuguese in the (grouped) positive emotions (amusement, pleasure, relief) combined, compared to the (grouped) negative ones (sadness, anger, fear) combined (**Supplemental** **Figure 1** and **Supplemental Table 1**).

### ***Response latency***

The pairwise comparison of emotion valence was statistically significant [*F*(1,60) = 47.65, *p* = < .001, *η_p_^2^* = 0.44, 95% CI [0.25 0.58], *d* = -6.00, **Supplemental Table 1**), showing that (grouped) negative emotions are recognized faster than (grouped) positive ones (**Figure 2** and **Supplemental Figure 1**).

The pairwise comparison of emotion valence and the interaction of emotion valence by nationality were not statistically significant (**Supplemental Table 1**).

## **2. Emotion authenticity task**

### ***Rating***

Additionally, we found that the (grouped) positive emotional vocalizations (amusement, pleasure, relief) were rated as more authentic than the negative ones (sadness, anger, fear) (*F*(1,61) = 57.69), *p* < .001, *η_p_^2^* = 0.49, 95% CI [0.30 0.61], *d* = 7.60; **Figure 2**, **Supplemental Figure 1** and **Supplemental Table 1**).

### ***Response latency***

Pairwise comparisons of emotion valence were not statistically significant (**Supplemental Table 1**).

# **Skin conductance response across all trials during the emotion recognition task**

## **1. Amplitude**

The main effect of emotion on the SCR amplitude was significant (*Wald 𝜒2* (5, *N* = 269) = 18.49, *p* = .002) and ordered from the highest to the lowest amplitude: anger, amusement, pleasure, sadness, fear, and relief (**Supplemental Figure 2**). Pairwise comparisons of emotions (**Supplemental Table 3**) showed significantly lower SCR amplitudes in relief than amusement, pleasure, sadness, and anger (**Supplemental Figure 2**). The main effect of nationality (*Wald 𝜒2* (1, *N* = 269) = .26, *p* = .611, *d* = -0.06) and interaction of emotion by nationality (*Wald 𝜒2* (5, *N* = 269) = 1.17, *p* = .948) were not significant.

## **2. Latency**

The main effects of nationality (*Wald 𝜒2* (1, *N* = 269) = .32, *p* = .571, *d* = 0.07) and emotion (*Wald 𝜒2* (1, *N* = 269) = 7.88, *p* = .163) and the interaction of emotion by nationality (*Wald 𝜒2* (1, *N* = 269) = 7.26, *p* = .202) on the latency of SCR were not significant.

## **3. Rise time**

The interaction of emotion by nationality on the SCR rise time was significant (*Wald 𝜒2* (1, *N* = 269) = 15.56, *p* = .008) so that the effect of nationality was significant in fear, but not in other emotions (i.e. amusement, pleasure, relief, sadness, and anger; **Supplemental Figure 2** and **Supplemental Table 3**). The main effect of nationality (*Wald 𝜒2* (1, *N* = 269) = .46, *p* = .498, *d* = -0.08) and emotion (*Wald 𝜒2* (1, *N* = 269) = 2.03, *p* = .846) on the SCR rise time were not significant.

## **4. Percentage**

The main effects of emotion on the SCR percentage was significant (*Wald 𝜒2* (5, *N* = 346) = 11.60, *p* = .041) and ordered from the highest to the lowest percentage: pleasure, relief, amusement, anger, sadness, and fear (**Supplemental Figure 2**). Pairwise comparisons of emotion (**Supplemental Table 3**) showed significantly lower SCR percentage in fear than in amusement, pleasure, and relief. The main effect of nationality (*Wald 𝜒2* (1, *N* = 346) = .79, p = .375, *d* = 0.09) and interaction emotion by nationality (*Wald 𝜒2* (5, *N* = 346) = 7.73, *p* = .172) on the SCR percentage did not reach statistical significance.

# **Confusion patterns and unbiased hit rates**

## **1. Emotion recognition task**

Unbiased hit rates for each emotion and their confusion patterns in percentage accuracy can be found in **Supplemental Table 4**.

**Supplemental Table 2-** Participants’ demographics of the sample used in the SCR analysis. Group comparisons between Guinea-Bissauan and Portuguese were performed with a two-sample t-test test for age and Chi square test or the Fisher’s Exact Test (if there are less than 5 cases in one of the tested groups) for all the other variables and marked with an asterisk if significant at a level of *p*-value < .05. **1.** personal history of drug addiction; **2.** use of recreational drugs in the last 6 months; **3.** consumes more than 28 units of alcohol per week (1 unit = ½ beer or 1 glass of wine); **4.** smokes more than 5 cigarettes per day; **5.** participant had a diagnosed mental illness in the past; **6.** participant had at least one family member with a diagnosed mental illness; n.a. not applicable.

|  | **Guinea-Bissau**  (*n* =32) | **Portugal**  (*n* =23) | **Group comparison**  **Guinea-Bissau vs. Portugal** |
| --- | --- | --- | --- |
| **Age** (years; mean ± standard deviation) | 25.8 ± 5.1 | 22.8 ± 5.8 | *t*(53) = 2.03, p = .047* |
| **Sex** (Male/Female) | 21/11 | 16/7 | χ^2^(1) = 0.09, p = .759 |
| **University education** (Yes/No) | 30/2 | 21/2 | p > .999 |
| **Heterosexual** (Yes/No) | 32/0 | 22/1 | p = .418 |
| **Handedness** (Right/Left) | 31/1 | 23/0 | p > .999 |
| **Drug addiction^1^** (Yes/No) | 0/23 | 0/31 | n.a. |
| **Drug use^2^** (Yes/No) | 0/32 | 5/18 | p = .010* |
| **Alcohol use^3^** (Yes/No) | 0/32 | 4/19 | p = .026* |
| **Tobacco use^4^** (Yes/No) | 1/31 | 1/22 | p > .999 |
| **Mental health problems^5^** (Yes/No) | 6/26 | 2/21 | p = .446 |
| **Family member with a mental illness^6^** (Yes/No) | 6/26 | 3/20 | p = .720 |

**Supplemental Table 3-** Post hoc pairwise comparisons for the main effects of emotion or ‘emotion x nationality’ interaction which were statistically significant across the physiological measures GEEs from the emotion recognition task. Statistically significant pairwise comparisons are marked with an asterisk (uncorrected p<.05). Effect size is represented with Cohen’s d (*d*) for post hoc pairwise comparisons from the GEEs.

|  | **SCR amplitude** | | | | **SCR percentage** | | | | **SCR rise time** | | | |
| --- | --- | --- | --- | --- | --- | --- | --- | --- | --- | --- | --- | --- |
|  | **emotion** | | | | **emotion** | | | | **emotion x nationality** | | | |
| **Comparisons** | **MD, 95% CI** | ***SE*** | **uncorrected *p*** | ***d*** | **MD, 95% CI** | ***SE*** | **uncorrected *p*** | ***d*** | **MD, 95% CI** | ***SE*** | **uncorrected *p*** | ***d*** |
| Amusement vs Pleasure | 0.01 [-0.06 0.08] | 0.04 | .764 | 0.05 | -1.16 [-4.41 2.10] | 1.66 | .487 | -0.07 | 0.02 [-0.05 0.10] | 0.04 | .520 | - |
| Amusement vs Relief | 0.13 [0.04 0.21] | 0.04 | .004* | 0.54 | 0.00 [-3.50 3.49] | 1.78 | .998 | 0.00 | -0.05 [-0.15 0.04] | 0.05 | .279 |  |
| Amusement vs Sadness | 0.02 [-0.06 0.09] | 0.04 | .658 | 0.08 | 2.19 [-2.33 6.70] | 2.30 | .342 | 0.13 | -0.01 [-0.11 0.10] | 0.05 | .910 |  |
| Amusement vs Anger | 0.00 [-0.07 0.07] | 0.04 | .990 | 0.00 | 1.43 [-1.88 4.74] | 1.69 | .398 | 0.09 | -0.01 [-0.09 0.06] | 0.04 | .736 |  |
| Amusement vs Fear | 0.04 [-0.03 0.12] | 0.04 | .266 | 0.20 | 3.44 [0.32 6.56] | 1.59 | .031* | 0.23 | -0.06 [-0.18 0.05] | 0.06 | .258 |  |
| Pleasure vs Relief | 0.12 [0.04 0.20] | 0.04 | .004* | 0.49 | 1.15 [-2.38 4.68] | 1.80 | .523 | 0.07 | 0.00 [-0.11 0.10] | 0.05 | .984 |  |
| Pleasure vs Sadness | 0.01 [-0.07 0.08] | 0.04 | .893 | 0.02 | 3.34 [-0.67 7.35] | 2.05 | .103 | 0.21 | 0.04 [-0.07 0.16] | 0.06 | .442 |  |
| Pleasure vs Anger | -0.01 [-0.08 0.06] | 0.04 | .751 | -0.05 | 2.58 [-0.48 5.64] | 1.56 | .098 | 0.17 | 0.04 [-0.05 0.13] | 0.05 | .413 |  |
| Pleasure vs Fear | 0.03 [-0.03 0.10] | 0.03 | .351 | 0.15 | 4.60 [1.66 7.53] | 1.50 | .002* | 0.32 | -0.01 [-0.14 0.11] | 0.06 | .819 |  |
| Relief vs Sadness | -0.11 [-0.18 -0.04] | 0.03 | .001* | -0.49 | 2.19 [-1.42 5.80] | 1.84 | .235 | 0.13 | -0.04 [-0.15 0.07] | 0.06 | .452 |  |
| Relief vs Anger | -0.13 [-0.20 -0.05] | 0.04 | .001* | -0.55 | 1.43 [-2.06 4.93] | 1.78 | .421 | 0.09 | -0.05 [-0.14 0.04] | 0.04 | .256 |  |
| Relief vs Fear | -0.09 [-0.17 0.00] | 0.05 | .056 | -0.39 | 3.44 [0.06 6.83] | 1.73 | .046* | 0.23 | -0.10 [-0.22 0.02] | 0.06 | .096 |  |
| Sadness vs Anger | -0.02 [-0.07 0.04] | 0.03 | .553 | -0.08 | -0.76 [-4.64 3.13] | 1.98 | .702 | -0.05 | 0.02 [-0.06 0.10] | 0.04 | .577 |  |
| Sadness vs Fear | 0.03 [-0.04 0.09] | 0.03 | .429 | 0.13 | 1.25 [-2.70 5.20] | 2.02 | .534 | 0.08 | -0.03 [-0.14 0.09] | 0.06 | .614 |  |
| Anger vs Fear | -0.04 [-0.11 0.02] | 0.03 | .199 | -0.21 | 2.01 [-0.84 4.86] | 1.45 | .167 | 0.15 | -0.09 [-0.20 0.03] | 0.06 | .157 |  |
| Amusement _GB – PT_ |  |  |  |  |  |  |  |  | 0.04 [-0.05 0.13] | 0.05 | .376 | -0.22 |
| Pleasure _GB – PT_ |  |  |  |  |  |  |  |  | 0.08 [-0.01 0.16] | 0.05 | .099 | 0.12 |
| Relief _GB – PT_ |  |  |  |  |  |  |  |  | -0.09 [-0.19 0.01] | 0.05 | .090 | -0.32 |
| Sadness _GB – PT_ |  |  |  |  |  |  |  |  | 0.03 [-0.08 0.14] | 0.05 | .583 | 0.19 |
| Anger _GB – PT_ |  |  |  |  |  |  |  |  | -0.03 [-0.12 0.05] | 0.04 | .434 | -0.09 |
| Fear _GB – PT_ |  |  |  |  |  |  |  |  | -0.13 [-0.25 -0.02] | 0.06 | .026* | 0.08 |

**Supplemental Table 4-** Recognition rates and their confusion patterns in percentage accuracy. Highlighted are the recognition rates of when the participant’s answer coincided with the intended nonverbal vocalization’s emotion. The unbiased hit rates for the Portuguese participants are 84.31% for amusement, 71.17% for pleasure, 65.81% for relief, 58.32% for fear, 78.61% for anger and 53.31% for sadness; whereas for the Guinea Bissauan participants the unbiased hit rates are 46.96% for amusement, 18.71% for pleasure, 38.53% for relief, 43.13% for fear, 46.75% for anger and 32.06% for sadness.

|  |  | **ANSWER** | | | | | | |
| --- | --- | --- | --- | --- | --- | --- | --- | --- |
| **STIMULI** | **CULTURE** | **Amusement** | **Pleasure** | **Relief** | **Fear** | **Anger** | **Sadness** | **Other** |
| **Amusement** | **PT** | 87.0 | 0 | 1.1 | 0.6 | 0.3 | 9.6 | 1.4 |
|  | **GB** | 62.9 | 11.4 | 1.6 | 1.9 | 0.7 | 16.0 | 5.2 |
| **Pleasure** | **PT** | 0.0 | 86.2 | 5.3 | 0.8 | 0 | 0.3 | 7.3 |
|  | **GB** | 6.1 | 37.4 | 43.5 | 3.1 | 1.6 | 2.8 | 5.4 |
| **Relief** | **PT** | 0.3 | 4.7 | 75.9 | 0.6 | 3.6 | 1.7 | 13.3 |
|  | **GB** | 1.6 | 9.8 | 72.2 | 5.1 | 1.9 | 2.3 | 7.0 |
| **Fear** | **PT** | 0 | 4.7 | 1.4 | 76.0 | 1.4 | 0.8 | 15.6 |
|  | **GB** | 2.6 | 4.0 | 4.7 | 70.6 | 1.6 | 7.1 | 9.4 |
| **Anger** | **PT** | 0.3 | 0 | 1.7 | 4.2 | 83.9 | 0 | 9.9 |
|  | **GB** | 8.4 | 5.6 | 5.6 | 11.2 | 53.9 | 2.6 | 12.6 |
| **Sadness** | **PT** | 2.3 | 8.8 | 2.3 | 17.3 | 0.3 | 63.7 | 5.4 |
|  | **GB** | 2.8 | 6.5 | 8.5 | 25.1 | 2.5 | 54.5 | 0 |


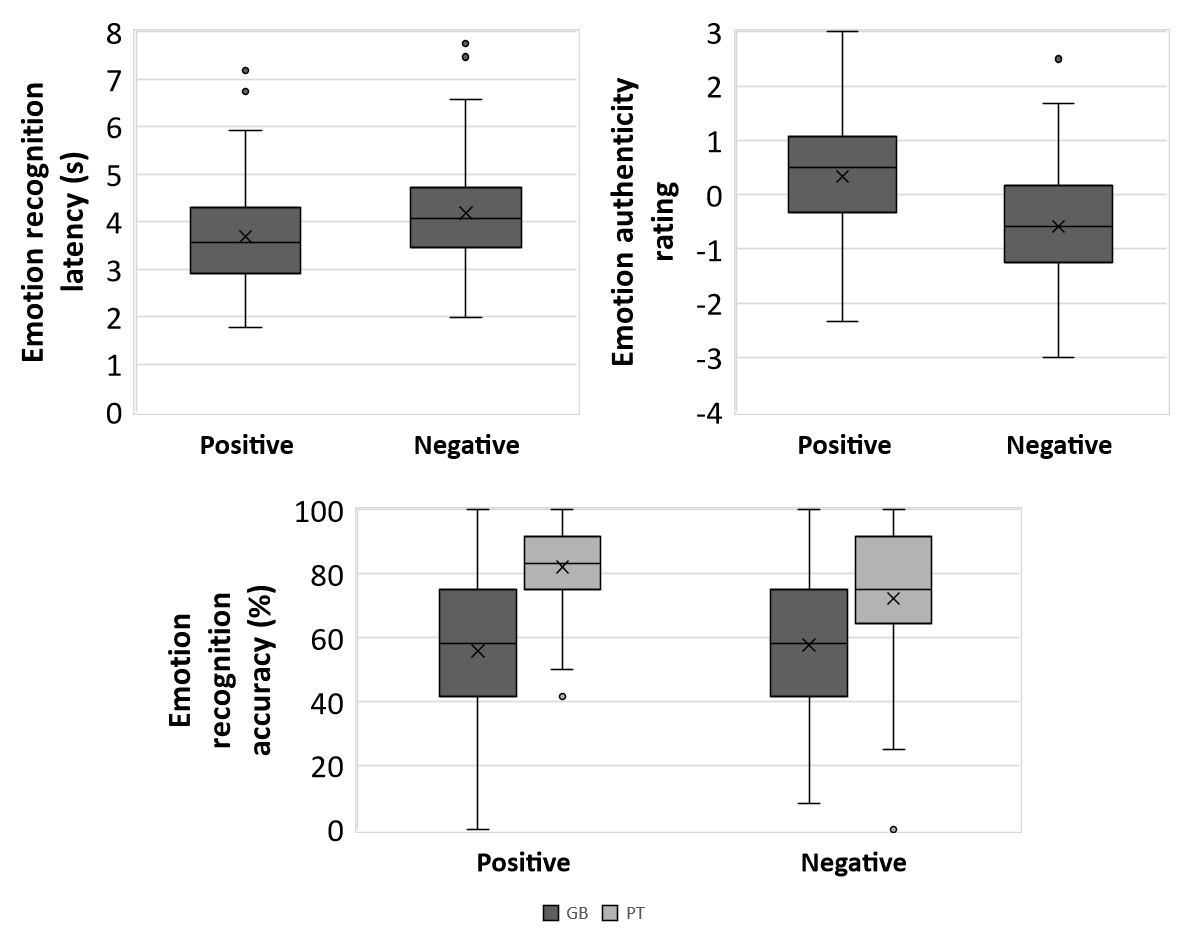


**Supplementary Figure 1-** Emotion valence effect [i.e. grouped positive emotions (amusement, pleasure, and relief) *versus* grouped negative emotions (sadness, anger, and fear)], with distribution (box plots) and mean values (cross) of the response latency of the emotion recognition task (top left), and the rating, of the authenticity recognition task (top right), for positive and negative emotions. The emotion valence by nationality effect is also represented for the emotion recognition accuracy (bottom). Only statistically significant emotion valence and emotion valence by nationality effects (at an FDR corrected p <.05) are shown, i.e. on the accuracy and response latency of the emotion recognition task and the rating of the emotion authenticity task.


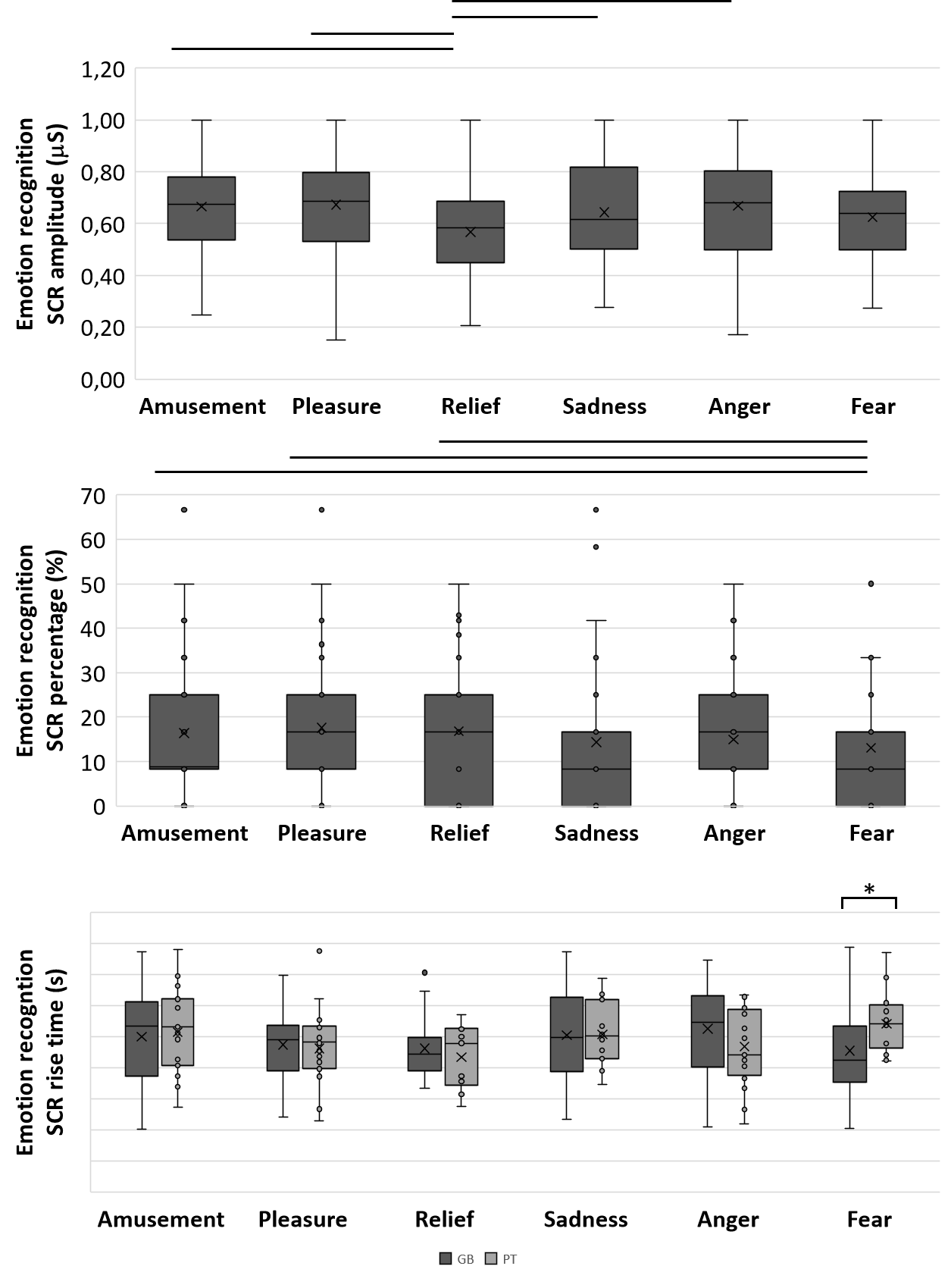


**Supplemental Figure 2-** Main effect of emotion (top and middle) and ‘emotion x nationality’ interaction effect (bottom), with distribution (box plots) and mean values (cross) of the SCR latency (top), percentage (middle) and rise time (bottom) across all trials of the emotion recognition task, for each emotion (amusement, pleasure, relief, sadness, anger, and fear) and nationality (Guinea-Bissauan – GB, and Portuguese – PT). Each line represents a statistically significant comparison (at an uncorrected p < .05) between emotions and significant comparisons between nationalities are marked with asterisks. Only statistically significant emotion main effects or ‘emotion x nationality’ interaction effects (at an uncorrected p <.05) are shown.
